# The influence of familiarity on memory for faces and mask wearing

**DOI:** 10.1186/s41235-022-00396-4

**Published:** 2022-05-15

**Authors:** Diana Kollenda, Benjamin de Haas

**Affiliations:** Experimental Psychology, Justus Liebig University, Giessen, Germany

**Keywords:** Face recognition, Face masks, Memory, Familiarity

## Abstract

During the COVID-19 pandemic, the wearing of face masks became mandatory in public areas or at workplaces in many countries. While offering protection, the coverage of large parts of our face (nose, mouth and chin) may have consequences for face recognition. This seems especially important in the context of contact tracing which can require memory of familiar and unfamiliar contacts and whether they were wearing a mask. In this study, we tested how well participants perform at remembering faces and whether they wore a mask, and if this depends on familiarity. Our results show that: (a) Participants remembered familiar faces better than unfamiliar ones, regardless of mask wearing. (b) Participants remembered unmasked faces better than masked faces, regardless of familiarity. (c) Participants were significantly worse at remembering whether an unfamiliar face was wearing a mask or not—even if they indicated remembering the face. (d) Participants showed a bias to indicate no memory of unfamiliar faces. (e) Participants showed a bias to indicate that unfamiliar faces wore a mask, even if they did not. In sum, it was harder to remember both, the identity of unfamiliar faces and whether they wore a mask. These findings have practical relevance for contact tracing and epidemic control.

**Supplementary Information:**

The online version contains supplementary material available at 10.1186/s41235-022-00396-4.

## Introduction

During the COVID-19 pandemic, non-pharmacological interventions were introduced in many countries (World Health Organization, 2021a, 2021b). This included mandatory face masks in public settings or workplaces and contact tracing. While protecting us and others (Howard et al., 2021), the coverage of large parts of the face (nose, mouth and chin) may have consequences for how we memorize and recognize faces. In the context of contact tracing, we may be asked to indicate which familiar and unfamiliar individuals we encountered during a given period and whether they wore a mask during these encounters. How good are we at remembering who we met and whether they wore a mask?

### The importance of familiarity

A key effect of face familiarity is that it leads to a predictable recognition enhancement for new images of an identity. Studies using recognition memory tasks have consistently shown that most people recognize familiar faces better than unfamiliar ones (for a review, see Johnston & Edmonds, 2009; Kramer et al., 2018; Young & Burton, 2017). While familiar face recognition is surprisingly robust against changes in viewing conditions, unfamiliar face recognition is more fragile and can be disrupted by even superficial changes of the particular image one is viewing (e.g., when the individual is caught in poor lighting) (Bruce, 1982; Hancock et al., 2000; Johnston & Edmonds, 2009).

The critical difference between familiar and unfamiliar face recognition might be that we have gained ‘stability from variation’ for familiar faces (Bruce, 1994): Repeated and highly diverse exposure to an individual’s face creates a representation of that particular face and its idiosyncratic variability (see also Bruce, 1982; Jenkins et al., 2011; Young & Burton, 2017). This robust mental representation might aid the recognition of a novel image of a known person, even if superficial changes occurred (Burton et al., 2016; Jenkins et al., 2011; Kramer et al., 2018). The situation is very different for unfamiliar faces (Bruce, 1982; Jenkins & Burton, 2011). If we have not seen a face before or have little experience with it, it is hard to tell whether differences in two images reflect the variability between different faces or within-person variability (cf. Burton et al., 2016). While decades of research have investigated the effects of familiarity on face recognition, less is known about the perceptual effects of face masks.

### The effect of face masks

In general, occluding parts of the face has large effects on recognition and matching performance. For instance, an early study by Terry (1994) demonstrated that the addition and removal of a beard as well as the removal of sunglasses impaired the recognition accuracy of faces. Furthermore, Nguyen and Pezdek (2017) investigated the effect that sunglasses (upper region of the face) and bandanas (lower region) have on participants’ recognition memory of unfamiliar faces. The accuracy was lowest when faces were wearing sunglasses, suggesting that especially the eye region provides important diagnostic information, independent of racial groups. However, both disguises had a detrimental effect on recognition performance in comparison with the no disguise condition (Nguyen & Pezdek, 2017). The importance of the mouth region for face identification was further stressed in a study by Mileva and Burton (2018). Their results suggested that especially open-mouthed smiles are useful for face identification. The authors argue that this type of smile provides idiosyncratic information about an identity (e.g., shape of the smile and teeth as well as wrinkles around the mouth). Furthermore, faces wearing ski-masks during an encoding stage were remembered less accurately in a subsequent recognition test (Manley et al., 2019).

When faces were partly occluded by masks, studies found a quantitative impairment of face recognition performance (Dhamecha et al., 2014; Marini et al., 2021) as well as qualitative changes in face processing (weaker inversion effect) (Freud et al., 2020). In addition, drastic changes in the appearance (e.g., disguise) seem to impair familiar and unfamiliar face perception (Noyes & Jenkins, 2019).

In line with the findings above, Noyes et al. (2021) and Carragher and Hancock (2020) found a detrimental effect of masks for face perception for both, familiar and unfamiliar faces. Noyes et al. (2021) used a matching task in which participants had to indicate whether unconcealed or concealed pairs of natural face images (i.e., one of two faces wore a mask) depict the same identity (a ‘match’) or two different people. They found a decrease in matching performance for familiar and particularly unfamiliar faces wearing masks. Similarly, Carragher and Hancock (2020) asked their participants to complete a matching task with three different conditions: (1) two faces were shown without masks, (2) one face was wearing a mask or (3) both faces were wearing masks. Interestingly, if at least one of the two faces was shown with a mask, participants were biased to accept familiar faces as ‘matches’ and to reject unfamiliar faces as ‘mismatches.’ This may reflect the lacking invariance of unfamiliar face recognition and point to observers ‘filling in’ occluded portions of familiar faces. However, both studies focused on matching performance and did not assess memory performance for faces and whether they wore a mask, both of which have practical relevance for epidemic control.

Taken together, partial face occlusion has detrimental effects on face perception and recognition. This may lead to diverging biases depending on familiarity, since the recognition of unfamiliar faces is less robust to superficial changes. Yet, it is unclear how masks affect our memory of familiar and unfamiliar faces and, in turn, how familiarity affects memory for mask wearing.

### Aim of this study

Here, we tested human short-term memory for familiar and unfamiliar identities and their wearing of masks in a series of online experiments. Specifically, we probed the effect of masks during an encoding stage on recognition performance and response biases for familiar and unfamiliar faces in a subsequent retrieval test. Similarly, we tested the ability and response biases for discriminating whether a previously seen familiar or unfamiliar face wore a mask or not.

In Experiment 1, we estimated the familiarity of faces in a standardized stimulus set for our study population. In Experiments 2, 3 and 4, separate samples each completed a study and a test phase. The study phase consisted of an attention task, in which participants were asked to indicate the gender of a presented face. The test phase consisted of a ‘surprise memory’ task for those faces. Experiments 2 and 4 tested the expected recognition advantage for familiar compared to unfamiliar faces for masked and unmasked faces. Experiments 3 and 4 tested the ability to remember whether unfamiliar and familiar faces wore a mask during the study phase. In addition, we explored related response biases as a function of familiarity.

## Methods

### Participants

We recruited all participants from Giessen University during April–July 2021 (14–16 months after mask wearing became common in Germany). They participated with their own devices and could choose between course credit and 8 EUR/h as compensation for their participation. All participants gave informed consent, and the study was performed in line with relevant guidelines and regulations and was approved by the local ethics review board (LEK-FB06) at Justus Liebig University Giessen.

### Stimuli and design

#### Face images

We collected two different frontal face images from 200 identities (100 females and 100 males) in the publicly available Celebrities in Frontal-Profile (CFP) dataset (Sengupta et al., 2016; Additional file 1: Figures S2.1–S2.6). These 200 identities were preselected from a pool of 500 identities based on their expected familiarity for our German study cohort, aiming for both extremes. Because these images were captured ‘in the wild,’ they vary widely on many factors including image quality, lighting conditions and facial expression. Overall, we ensured that the images selected for the stimulus pool captured the face in near frontal orientation and tend to show a smile or neutral expression. In all four (online) experiments, the experimental software presented the images in color, at the screen center and rescaled to a physical height of 4.7 cm (1.85 inches) on the respective participant’s screen, keeping the original aspect ratio. A white space was added to the top, bottom and sides as needed. The minimum screen size for participation was 12 inches (i.e., laptops or desktop screens, but no tablets or smartphones).

#### Face masks

We used face masks in Experiments 3 and 4. Images of face masks were collected online and superimposed on the face images using GIMP (version 2.10.24), in a way that aimed to emulate the wearing of face masks in real life, from the middle of the nose to below the chin (see Fig. 3). We used different face masks in order to match the face contour most realistically. All images of masked faces are provided with the data and code accompanying this paper and may be downloaded and used by other researchers [https://osf.io/fcxaj/].

### Procedure

We conducted all experiments online using lab.js (Henninger et al., 2020), which is compatible with most web browsers, operating systems and devices. We asked participants to perform the experiment in an upright sitting posture. Before starting the experiment, participants gave informed consent, created a participant code, and provided demographic information (age, gender). In Experiment 1, participants were asked to rate the familiarity of 400 images corresponding to 200 identities (for details see below). Experiments 2, 3 and 4 consisted of two phases: The study phase was followed by the test phase after a short break. In both phases, the sequential presentation of familiar and unfamiliar faces and (if applicable) masked and unmasked faces was intermixed and random. The study phase was structured the same in all three experiments and included an attention task. Participants were asked to indicate to which gender they assign a certain face (1 = male, 2 = female or 3 = nonbinary). The test phase included a ‘surprise memory’ task in which we asked participants to indicate their memory for the presence of certain faces or their wearing of masks in the study phase (for details see individual experiments, below and find the original German instructions in the Additional file 1: Material S1). Note that we always presented different frontal photographs of a given identity in the study and test phases.

### Analysis

We analyzed the data using R (version 4.0.3; R Core Team, 2020) and plotted results using the ‘ggplot2’ package (Wickham, 2016).

For Experiment 1, we computed intra-class correlation coefficients using the ‘irr’ package (Gamer et al., 2019) and interpreted the results following the guidelines given by Koo and Li (2016).

For Experiments 2, 3 and 4, we computed the signal detection measures d′ (‘d-prime’) and *c* (criterion) for each participant using the ‘psycho’ package (Makowski, 2018), in order to get independent estimates of sensitivity and response biases (Macmillan & Creelman, 2004; Stanislaw & Todorov, 1999). In accordance with signal detection theory (SDT), the signal was defined as ‘face present in the study phase’ or ‘face wore a mask in the study phase.’ Thus, we considered hits (correctly responding to have seen a face or a mask), false alarms (incorrectly responding to have seen a face or a mask), misses (incorrectly responding, not having seen a face or a mask) and correct rejections (correctly responding, not having seen a face or a mask) for our computations. Note that when the signal was ‘presence of a face in the study phase,’ we used lures that were only shown in the test phase to calculate the false alarm rate (see below for details).

*d*′ can be computed by subtracting the *z* score that corresponds to the false alarm rate from the *z* score that corresponds to the hit rate:

1 $$d^{\prime } = z\left( H \right){-}z\left( F \right)$$

The hit rate (*H*) is the probability of a hit and computed as

2 $$H = n_{{\text{H}}} /\left( {n_{{\text{H}}} + \, n_{{{\text{miss}}}} } \right)$$

where *n*_H_ corresponds to the number of hits and *n*_miss_ to the number of misses. The false alarm rate (*F*) is the probability of a false alarm and computed as

3 $$F = n_{{\text{F}}} /\left( {n_{{\text{F}}} + \, n_{{{\text{cr}}}} } \right)$$

where ${n}_{\mathrm{F}}$ is the number of false alarms and *n*_cr_ is the number of correct rejections. Since the *z* score takes on infinite values when either *F* or *H* is equal to zero or one (Hautus, 1995), a correction was used in these cases. The adjusted formulas used by the ‘psycho’ package are:

4 $$H_{{{\text{adjusted}}}} = \, \left( {n_{{\text{H}}} + \, 0.5} \right)/\left( {n_{{\text{H}}} + \, n_{{{\text{miss}}}} + \, 1} \right)$$

and

5 $$F_{{{\text{adjusted}}}} = \left( {n_{{\text{F}}} + \, 0.5} \right)/\left( {n_{{\text{F}}} + \, n_{{{\text{cr}}}} + \, 1} \right)$$

*c* was computed by building the negative sum of the *z* score that corresponds to the hit rate and the *z* score that corresponds to the false alarm rate and divide it by two:

6 $$c = - \left( {z\left( H \right) + z\left( F \right)} \right)/2$$

For both signal detection measures, we performed 2 × 2 repeated measures ANOVAs in Experiment 4 by using the ‘ez’ package (Lawrence, 2016). Furthermore, we computed pairwise t tests using the ‘rstatix’ package (Kassambara, 2020) and adjusted two-sided *p* values for multiple comparisons (indicated with *p*_adj_) using the ‘holm’ correction method (Holm, 1979). The alpha level for all statistical analyses was set at 0.05.

#### Control analysis

In Experiment 2, we tested whether familiar faces are remembered better than unfamiliar ones in a surprise memory task. We measured performance by using hit rates to unfamiliar or familiar target faces (see Experiment 1 for details; 0–25% and 75–100% percentile in familiarity, respectively) and false alarms computed on two different types of lures that were only shown in the test phase: unfamiliar lures (25–50% percentile in familiarity) and familiar lures (50–75% percentile in familiarity). This classification may favor performance on familiar faces if participants rely on a ‘familiarity heuristic’ to complete the task. Specifically, for the set of familiar faces, the familiar lures are less familiar than the targets, while for unfamiliar faces lures were *more* familiar than targets. So a familiarity heuristic should lead to higher hit rates and lower false alarm rates in the familiar condition, but lower hit rates and higher false alarm rates in the unfamiliar condition. To control for this confound, we first regressed the frequency of ‘yes’ responses (likely seen or seen) onto familiarity across all lure trials. The slope of this function serves as an estimate of the effect size of the hypothesized familiarity heuristic, i.e., how much more likely participants were to (falsely) answer likely seen or seen for more familiar lures, Specifically, the slope of the function indicates the expected number of additional ‘yes’ responses per additional point on the familiarity rating scale and 100 trials (i.e., the total number of lure trials). We then multiplied this number with the average familiarity difference between familiar lures and targets and the number of trials in the familiar condition (50 trials, i.e., factor 0.5) to estimate the additional hits and lacking false alarms in the familiar condition due to this confound. Converting the respective number of hits to misses and correct rejections to false alarms for each individual allowed us to estimate a downward corrected d' for the familiar condition. Similarly, we multiplied the estimated effect size of the familiarity heuristic with the average familiarity difference between unfamiliar lures and unfamiliar targets to convert the respective number of misses to hits and false alarms to correct rejections, thus upward correcting the $d^{\prime }$ for the unfamiliar condition. Finally, we tested the robustness of the familiarity advantage, by comparing the corrected $d^{\prime }$ between conditions. We also applied the same correction to data from Experiment 4, separately for each cell of the mask by familiarity condition (25 trials per participant each).

## Results

### Experiment 1

**Fig. 1:**
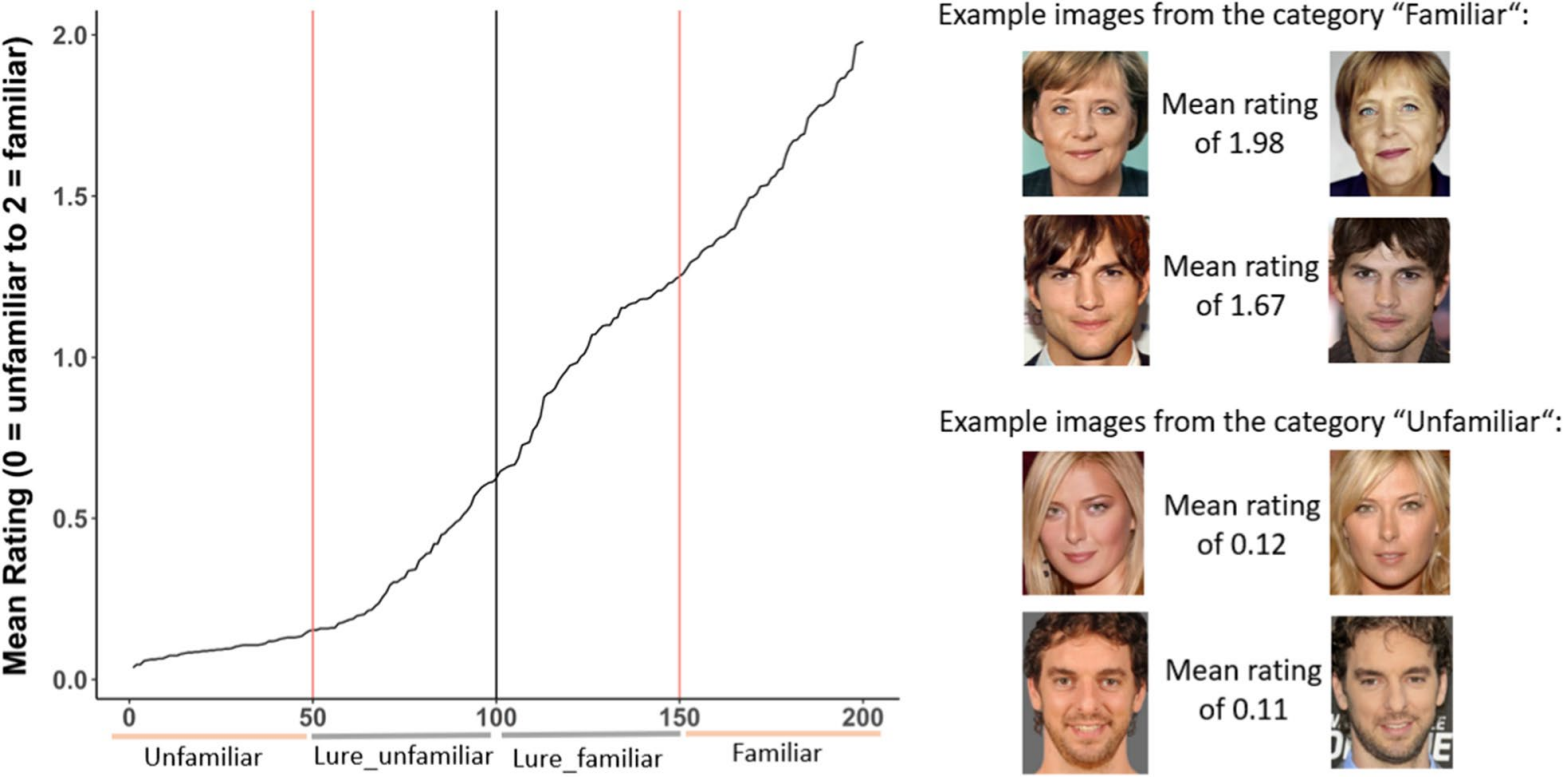
Familiarity rating for faces of 200 identities. *Note.* The plot on the left shows the mean rating of familiarity for a given identity. In total, 200 identities were rated based on two different frontal pictures. The right side of the figure illustrates the mean rating across all participants for four sample identities

In total, 215 participants (54 males, 157 females, 3 nonbinary, age *M* = 25.21, *SD* = 7.5), recruited from Giessen University, took part in Experiment 1. In order to control which faces are familiar or unfamiliar to our test population, we let participants rate the familiarity of 200 faces selected from the CFP dataset (Sengupta et al., 2016) (see Additional file 1: Material S2). In total, there were 400 trials and the experiment lasted about 25 min. Each trial consisted of a central fixation cross, which lasted for 700 ms, and a subsequent image presentation. Face images were shown one at a time until participants responded by key press whether they do not know a face (key 1, coded as 0), they know a face but cannot name it (key 2, coded as 1) or they know a face and can name it (key 3, coded as 2). All images were presented in a pseudo-random order, which was different for each participant. Note that we presented two frontal images per identity and calculated the mean response of these two images (see Fig. 1 for examples).

#### Results and discussion

Our results suggest that the 50 least familiar faces were unknown to the majority of participants. Within the top 50, there is some variance, but the difference between the 50 least and most familiar faces was very high (Fig. 1) and that between the second and third quartile is still substantial. Thus, we clustered the stimulus pool into four different categories with 50 images each: Unfamiliar (bottom quartile; *M* = 0.1; *SD* = 0.36), Lure_unfamiliar (second quartile; *M* = 0.35; *SD* = 0.65), Lure_familiar (third quartile; *M* = 1; *SD* = 0.91) and Familiar (top quartile; *M* = 1.59; *SD* = 0.71).

To assess the generalizability of familiarity ratings, we computed the intra-class correlation coefficient. There was an excellent absolute agreement, as revealed by a two-way random effects model with ‘average rater’ unit: ICC = 0.995 with 95% confidence interval = 0.994–0.996. This analysis indicates that the mean familiarity ratings for our stimuli can be expected to reproduce for an independent sample with high precision.

### Experiment 2—surprise memory task for identity

The aim of Experiment 2 was to investigate if participants remember familiar faces better than unfamiliar ones. We recruited 44 healthy participants (14 males, 30 females; age *M* = 28.43, *SD* = 10.69) from Giessen University.

The total duration of the experiment was roughly 25 min. Each trial consisted of a central fixation cross, which lasted for 700 ms, and a subsequent image presentation. Each image was shown one at a time until participants responded by key press. In the study phase, we presented one frontal image for each identity of the categories Familiar and Unfamiliar (50 each, i.e., 100 trials). In the test phase, we presented the second frontal image from the same identities, intermixed with Lure_familiar and Lure_unfamiliar stimuli that were not shown in the study phase (50 each for all four categories, i.e., 200 trials). All trials were presented in a pseudo-random order, and participants were asked to indicate by keypress if they had seen the faces in the first half of the experiment already. Rating responses could be − 2 (key 1; ‘No, not seen’); − 1 (key 2; ‘Likely not seen’); 1 (key 3; ‘Likely seen’); 2 (key 4; ‘Yes, seen’). For the SDT analysis, we grouped the answers, with − 2 and − 1 corresponding to ‘no’ and + 1 and + 2 to ‘yes.’

#### Results and discussion

**Fig. 2:**
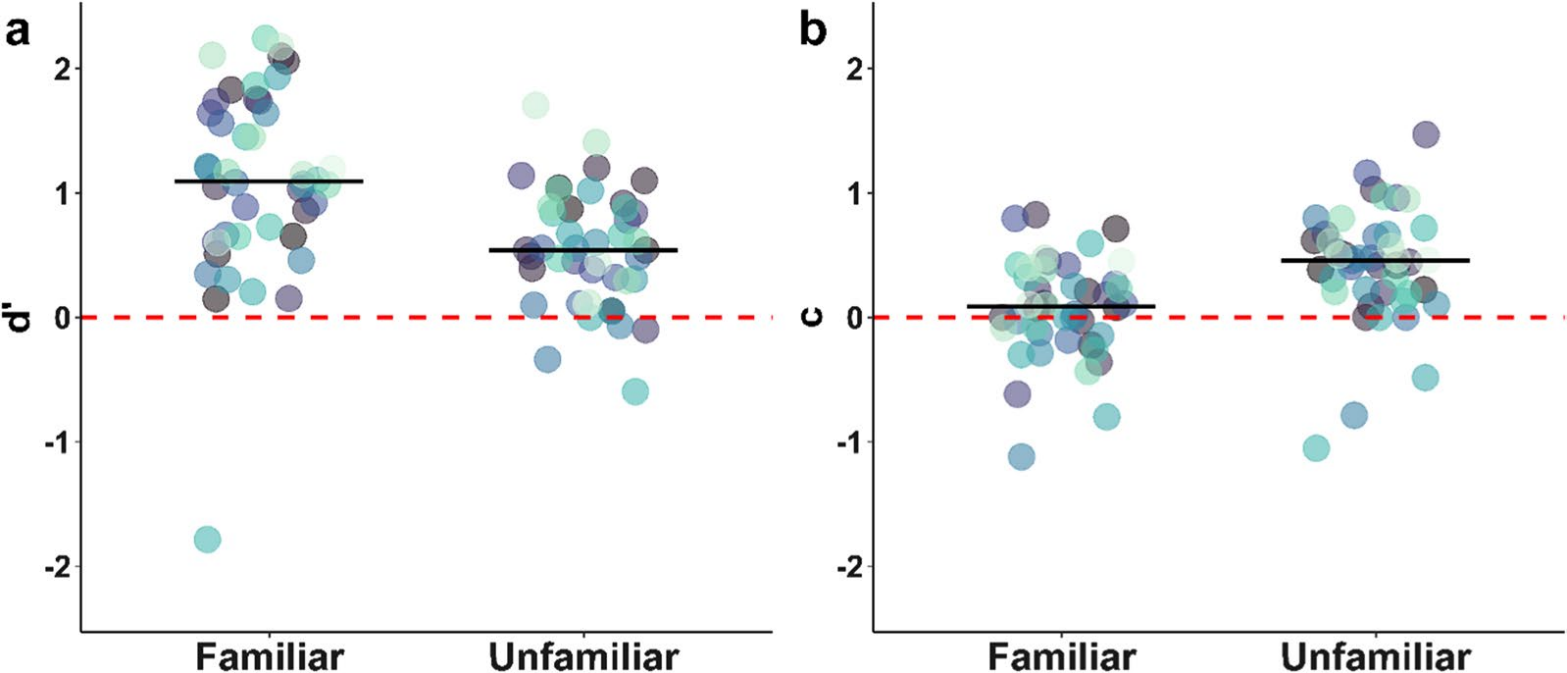
Sensitivity ($d^{\prime }$) and response bias (*c*) for identity recognition, depending on the familiarity of faces. *Note.* Black horizontal lines indicate median values. Same colors indicate data points from the same participant. Each data point shows the sensitivity ($d^{\prime }$; panel **a**) or response bias (*c*; panel **b**) of a single participant indicating whether they saw a given face in the study phase, separately for familiar and unfamiliar faces. **a** Advantage for remembering familiar faces. Higher values of $d^{\prime }$ indicate better performance. A value of zero indicates ‘guessing.’ **b** Stronger conservative bias for unfamiliar faces. Values greater than zero indicate a conservative bias (tendency to answer ‘no’), and values lower than zero indicate a liberal bias (tendency to answer ‘yes’)

In total, participants had a mean (SD) hit rate of 34.33 (8.0) for familiar faces and 21.86 (7.35) for unfamiliar faces. They had a mean (SD) false alarm rate of 13.81 (8.34) for familiar face lures and 12.51 (7.75) for unfamiliar face lures. Participants showed a significant memory advantage (higher sensitivity) for familiar faces ($d^{\prime }$ = mean ± standard error of the mean; $d^{\prime }$ = 1.10 ± 0.11) compared to unfamiliar faces ($d^{\prime }$ = 0.54 ± 0.07), *t*(43) = 7.62, *p* < 0.001, *d* = 1.15. The effect was even stronger, when excluding one outlier, which performed highly significantly *below* chance (and maybe did not understand the instruction) (Fig. 2a). Without the outlier, the test statistic corresponded to *t*(42) = 9.57, *p* < 0.001, *d* = 1.46 ($d^{\prime }$ = 1.17 ± 0.09 and 0.57 ± 0.07 for familiar and unfamiliar faces, respectively). Participants also showed a significantly stronger conservative bias (tendency to indicate no memory) for unfamiliar faces (*c* = 0.41 ± 0.07) than familiar faces (*c* = 0.07 ± 0.06), *t*(43) =  − 5.02, *p* < 0.001, *d* =  − 0.76 (Fig. 2b). After excluding the one outlier, this test statistic corresponded to *t*(42) =  − 7.54, *p* < 0.001, *d* =  − 1.15 (*c* = 0.45 ± 0.06 for unfamiliar and 0.06 ± 0.06 for familiar faces). The conservative bias for unfamiliar faces was significantly different from zero, *t*(42) = 7.27, *p* < 0.001, *d* = 1.11, whereas there was no significant bias for familiar faces, *t*(42) = 0.98, *p* = 0.33, *d* = 0.15. Note that these results were robust, even if the possible confounding effect of a 'familiarity heuristic' was removed (Additional file 1: Analysis S3).

### Experiment 3—surprise memory task for masks

**Fig. 3:**
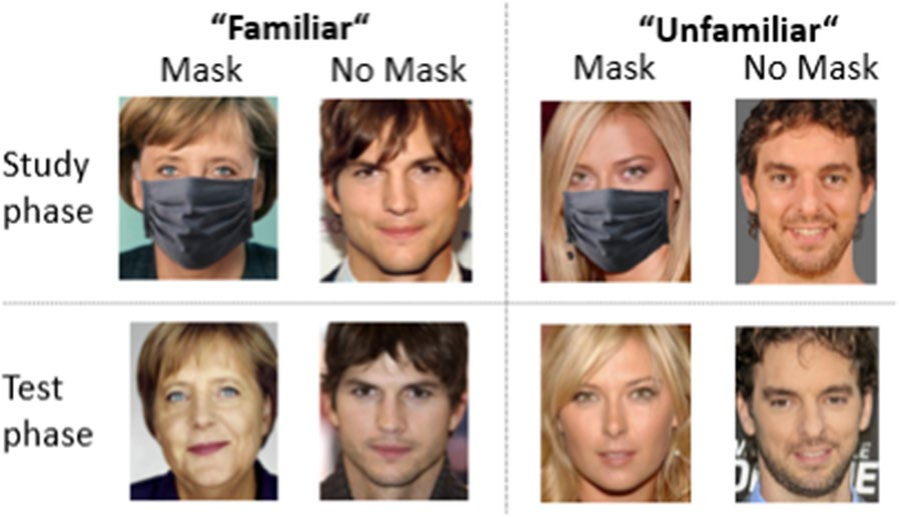
Study design in Experiment 4. *Note.* The experiment consisted of a study phase, in which half of the familiar and unfamiliar faces (25 each) were shown with a mask and the other half without. In the subsequent test phase, all faces were shown without masks. We used two different frontal pictures of the same identity in the two phases

The rationale for Experiment 3 was to investigate if the ability to remember whether a face was wearing a mask is modulated by face familiarity. Thus, we presented familiar and unfamiliar faces with and without masks during the study phase (see Fig. 3).

In total, we tested 123 participants (31 males, 91 females, 1 nonbinary; age *M* = 27.15, *SD* = 9.72) from Giessen University. The total duration of the experiment was roughly 15 min. Each trial consisted of a central fixation cross, which lasted for 700 ms, and a subsequent image presentation. Each image was shown one at a time until participants responded by key press. In the study phase (100 trials), half of the familiar and unfamiliar faces (i.e., 25 faces each) were presented with a mask. The other half was not wearing a mask. We counterbalanced which identities were shown with a mask or without across all participants so that across participants, every familiar and unfamiliar face was shown with a mask and without a mask (cross-design).

In the test phase of the experiment (100 images), we presented the second frontal picture of each identity. Participants were asked to rate whether they had seen the respective face wearing a mask in the first half of the experiment. Responses could be − 2 (key 1; ‘No, not seen with a mask’); − 1 (key 2; ‘Likely not seen with a mask’); 1 (key 3; ‘Likely seen with a mask’); and 2 (key 4; ‘Yes, seen with a mask’). For the SDT analysis, we grouped the answers, with − 2 and − 1 corresponding to ‘no’ and + 1 and + 2 to ‘yes.’

#### Results and discussion

**Fig. 4:**
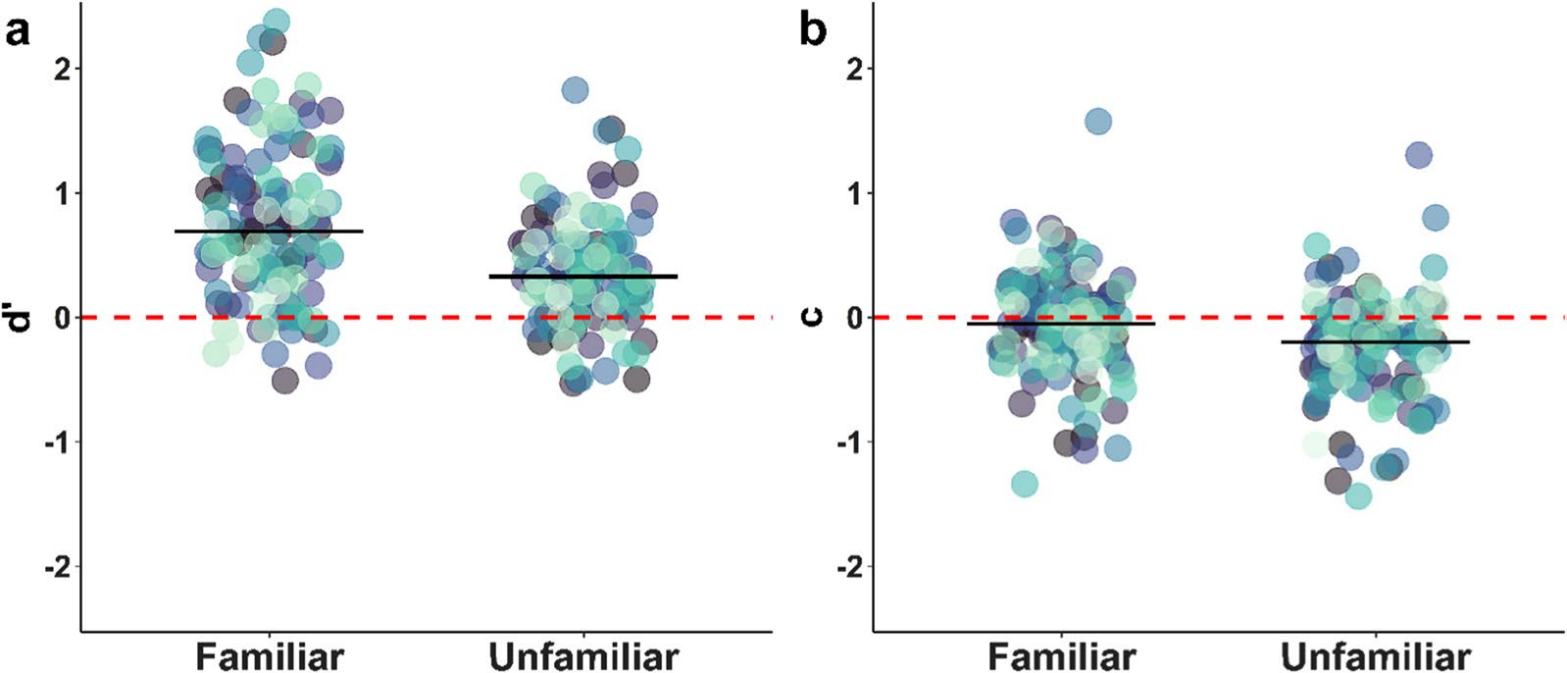
Sensitivity ($d^{\prime }$) and response bias (*c*) for mask memory, depending on the familiarity of faces. *Note.* Black horizontal lines indicate median values. Same colors indicate data points from the same participant. Each data point shows the sensitivity ($d^{\prime }$; panel **a**) or response bias (*c*; panel **b**) of a single participant indicating whether a given face wore a mask in the study phase, separately for familiar and unfamiliar faces. **a** Advantage for remembering whether a familiar face wore a mask. Higher values of $d^{\prime }$ indicate better performance. A value of zero indicates ‘guessing.’ **b** Stronger liberal bias for indicating unfamiliar faces wore a mask. Values greater than zero indicate a conservative bias (tendency to answer ‘no’), and values lower than zero indicate a liberal bias (tendency to answer ‘yes’)

Participants showed a significant advantage for remembering whether a familiar face wore a mask ($d^{\prime }$ = 0.75 ± 0.05) over mask memory for unfamiliar faces ($d^{\prime }$ = 0.36 ± 0.04), *t*(122) = 7.11, *p* < 0.001, *d* = 0.64. (Fig. 4a). They also showed a significantly more liberal bias for unfamiliar faces (tendency to indicate the face wore a mask; *c* =  − 0.26 ± 0.04) compared to familiar faces (*c* =  − 0.07 ± 0.04), *t*(122) = 5.05, *p* < 0.001, *d* = 0.46 (Fig. 4b). For unfamiliar faces, *c* indicated a significant liberal bias *t*(122) =  − 6.7, *p* < 0.001, *d* =  − 0.6, whereas *c* for familiar faces only showed a nonsignificant tendency for a liberal bias, *t*(122) =  − 1.78, *p* = 0.078, *d* =  − 0.16. Note that in Additional file 1: Analysis S4, we report an additional logistic mixed-effects model in which the probability of a participant responding with 1 or 2 (i.e., likely yes or yes) is estimated as a function of the fixed effects of mask condition and familiarity rating as well as the interaction between them. This analysis replicated the pattern of results shown here, with significant effects for mask condition and the interaction between familiarity rating and mask condition. We opted to present signal detection results in the main text since familiarity ratings were on an ordinal scale truncated at [0;2] (see Experiment 1 for details), and we explicitly aimed for a bimodal distribution of familiarity when selecting our stimuli, rendering our experimental design better suited for this categorical analysis.

### Experiment 4—surprise memory task for identity and masks

The motivation for Experiment 4 was to test the effects of masks in the study phase on identity recognition and to further explore the effect of familiarity on mask memory and its potential dependence on identity recognition. Therefore, we designed a dual task in which we tested identity and mask memory for the same set of stimuli in the same sample of participants.

We recruited 64 healthy participants (16 males, 47 females, 1 nonbinary; *M* = 24.86, *SD* = 5.76) from Giessen University. The total duration of the experiment was 25 min. Each trial consisted of a central fixation cross, which lasted for 700 ms, and a subsequent image presentation. Each image was shown one at a time and until participants answered the questions regarding the image by key press. In the study phase of the experiment (100 trials), faces of familiar and unfamiliar identities were presented either with a mask or without (see Experiment 3).

In the test phase (200 trials), we presented the second frontal image of each identity shown in the study phase intermixed with lure images (Lure_familiar and Lure_unfamiliar), sequentially and in a pseudo-random order, all without masks. We asked participants to answer two questions per image. Question 1 was: ‘Did you see this face in the first part of the experiment?’. Responses could be − 2 (key 1; ‘No, not seen’); − 1 (key 2; ‘Likely not seen’); 1 (key 3; ‘Likely seen’); and 2 (key 4; ‘Yes, seen’). For the SDT analysis, we grouped the answers, with − 2 and − 1 corresponding to ‘no’ (not seen) and + 1 and + 2 to ‘yes’ (seen). Question 2 was: ‘Suppose this face was presented in the first part of the experiment. Was it shown with or without a mask?’. Responses could be − 2 (key 1; ‘I have seen the face without mask’); − 1 (key 2; ‘Likely seen without a mask’); 1 (key 3; ‘Likely seen with a mask’); and 2 (key 4; ‘I have seen the face with a mask’). For the SDT analysis, we grouped the answers, with − 2 and − 1 corresponding to ‘no’ and + 1 and + 2 to ‘yes.’

Additionally, we analyzed responses to Question 2 (mask) grouped by responses to Question 1 (face seen vs. unseen). We refer to this analysis as ‘rating’ based. For this analysis, we only included participants with at least five trials in each condition (e.g., familiar faces without mask rated as seen). This led to the exclusion of 33 participants, leaving 31 participants (9 males, 22 females; *M* = 23.74, *SD* = 4.3) for this analysis.

#### Results and discussion

First, we analyzed answers to Question 1 (‘Did you see the face in the first part of the experiment?’). When faces wore masks in the study phase, participants had a mean (SD) hit rate of 15.16 (4.67) for familiar faces and 8.98 (5.02) for unfamiliar faces. When they were unmasked, participants had a mean (SD) hit rate of 17.31 (4.78) for familiar faces and 10.94 (4.84) for unfamiliar faces. They had a mean (SD) false alarm rate of 16.73 (9.2) for familiar face lures and 14.8 (9.64) for unfamiliar face lures. Descriptive statistics for $d^{\prime }$ and *c* are reported in Table 1. We conducted a repeated-measured ANOVA with two factors, familiarity (familiar vs. unfamiliar) and presence of a mask (mask vs. no mask) in the study phase for the measures sensitivity ($d^{\prime }$) and response bias (*c*), respectively. For both measures of $d^{\prime }$ and *c*, the main effects of familiarity and mask condition were significant, while the interaction between both factors was not (see Table 2).

**Table 1:** Memory for face identities (Question 1)

	Sensitivity ($d^{\prime }$)		Response bias (c)	
	Familiar	Unfamiliar	Familiar	Unfamiliar
Mask	0.75 (0.07)	0.21 (0.05)	0.08 (0.06)	0.50 (0.07)
No mask	1.02 (0.09)	0.44 (0.07)	− 0.05 (0.06)	0.39 (0.07)

Descriptive statistics [mean(standard error of the mean)] for the measures sensitivity ($d^{\prime }$) and criterion (*c*)

Results are shown separately for familiarity and the presence of a mask in the study phase.

**Table 2:** Memory for face identities (Question 1)

	Sensitivity ($d^{\prime }$)	Response bias (*c*)
Familiarity	*F*(1,63) = 85.39, *p* < .001*, $\eta_{p}^{2}$ = .19	*F*(1,63) = 82.98, *p* < .001*, $\eta_{p}^{2}$ = .15
Mask condition	*F*(1,63) = 39.18, *p* < .001*, $\eta_{p}^{2}$ = .04	*F*(1,63) = 39.18, *p* < .001*, $\eta_{p}^{2}$ = .01
Interaction	*F*(1,63) = 0.37, *p* = .547, $\eta_{p}^{2}$ = .00	*F*(1,63) = 0.37, *p* = .547, $\eta_{p}^{2}$ = .00

Repeated measures ANOVA for sensitivity ($d^{\prime }$) and response bias (*c*)

*Marks statistically significant results.

**Fig. 5:**
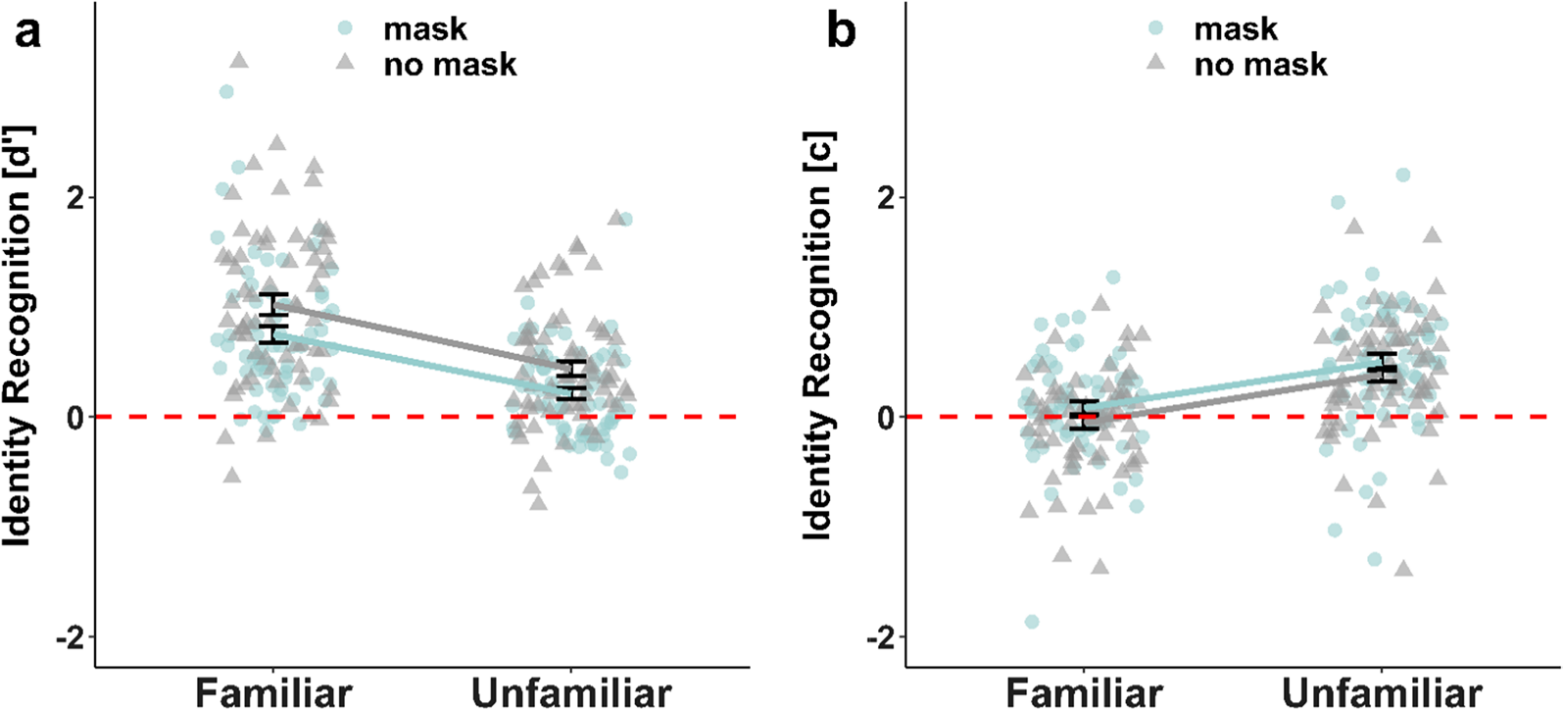
Sensitivity ($d^{\prime }$) and response bias (*c*) for identity recognition (Question 1). *Note.* Error bars indicate standard errors. Each data point shows the sensitivity ($d^{\prime }$; panel **a**) or response bias (*c*; panel **b**) of a single participant indicating whether they saw a face in the study phase, separately for familiar and unfamiliar faces and faces that wore a mask (blue dots) or not (gray dots) in the study phase. **a** Main effects of familiarity and masks on recognition sensitivity for faces. Higher values of $d^{\prime }$ indicate better performance. A value of zero indicates ‘guessing.’ **b** Main effects of familiarity and masks on recognition bias for faces. Values greater than zero indicate a conservative bias (tendency to answer ‘no’) and values lower than zero indicate a liberal bias (tendency to answer ‘yes’)

Using paired t tests, participants showed a significant memory advantage for familiar compared to unfamiliar faces, *t*(63) = 9.24, *p*_*adj*_ < 0.001, *d* = 1.16, and for unmasked compared to masked faces, *t*(63) =  − 6.26, *p*_*adj*_ < 0.001, *d* =  − 0.78 (Fig. 5a). Participants also showed a more conservative bias (tendency to indicate no memory) for unfamiliar compared to familiar faces, *t*(63) =  − 9.11, *p*_*adj*_ < 0.001, *d* =  − 1.14, which was significantly different from zero, *t*(63) = 6.49, *p*_*adj*_ < 0.001, *d* = 0.81. For familiar faces, *c* did not differ from zero, *t*(63) = 0.28, *p*_*adj*_ = 0.783, *d* = 0.03. Furthermore, participants showed a more conservative bias for faces with masks compared to faces without masks, *t*(63) = 6.26, *p*_*adj*_ < 0.001, *d* = 0.78 (Fig. 5b). Even though the interaction was not significant, we computed planned t test that allowed us to compare our results with a previous report regarding matching performance when two faces wore no mask or one out of two wore a mask (cf. Carragher & Hancock, 2020). There was a more conservative bias for unfamiliar faces with masks than without, *t*(63) = 4.33, *p*_*adj*_ < 0.001, *d* = 0.54, but participants showed a conservative bias for both, unfamiliar faces with, *t*(63) = 6.82, *p*_*adj*_ < 0.001, *d* = 0.85, and without masks, *t*(63) = 5.9, *p*_*adj*_ < 0.001, *d* = 0.74. Note that these results were robust, even if the possible confounding effect of a 'familiarity heuristic' was removed (Additional file 1: Analysis S5).

Regarding Question 2 (‘Suppose this face was presented in the first part of the experiment. Was it shown with or without a mask?’), descriptive statistics for $d^{\prime }$ and *c* are reported in Table 3. We performed repeated measures 2 × 2 ANOVAs for the measures $d^{\prime }$ and *c*, respectively. The factors were familiarity (familiar vs. unfamiliar) and rating for faces (seen vs. not seen). For $d^{\prime }$, the main effect of rating was significant as well as the interaction between both factors, familiarity and rating (see Table 4). For *c*, neither the main effects of familiarity and rating nor the interaction between both factors was significant (see Table 4).

**Table 3:** Memory for masks (Question 2)

	Sensitivity ($d^{\prime }$)		Response bias (*c*)	
	Familiar	Unfamiliar	Familiar	Unfamiliar
Rated as seen	0.65 (0.14)	− 0.03 (0.12)	0.16 (0.09)	0.09 (0.12)
Rated as not seen	− 0.18 (0.14)	0.17 (0.07)	− 0.08 (0.20)	− 0.02 (0.22)

Descriptive statistics [mean(standard error of the mean)] for the measures sensitivity ($d^{\prime }$) and response bias (*c*)

Results are shown separately for familiarity and cases in which participants rated the faces as seen or not seen in the study phase (Question 1).

**Table 4:** Memory for masks (Question 2)

	Sensitivity ($d^{\prime }$)	Response bias (*c*)
Familiarity	*F*(1, 30) = 2.67, *p* = .112, $\eta_{p}^{2}$ = .02	*F*(1, 30) = 0.0, *p* = .953, $\eta_{p}^{2}$ = .00
Rating	*F*(1, 30) = 7.87, *p* = .009*, $\eta_{p}^{2}$ = .07	*F*(1, 30) = 0.57, *p* = .455, $\eta_{p}^{2}$ = .01
Interaction	*F*(1, 30) = 21.91, *p* < .001*, $\eta_{p}^{2}$ = .16	*F*(1, 30) = 1.48, *p* = .233, $\eta_{p}^{2} = \, .00$

Repeated measures ANOVA for sensitivity ($d^{\prime }$) and response bias (*c*)

*Marks statistically significant results.

**Fig. 6:**
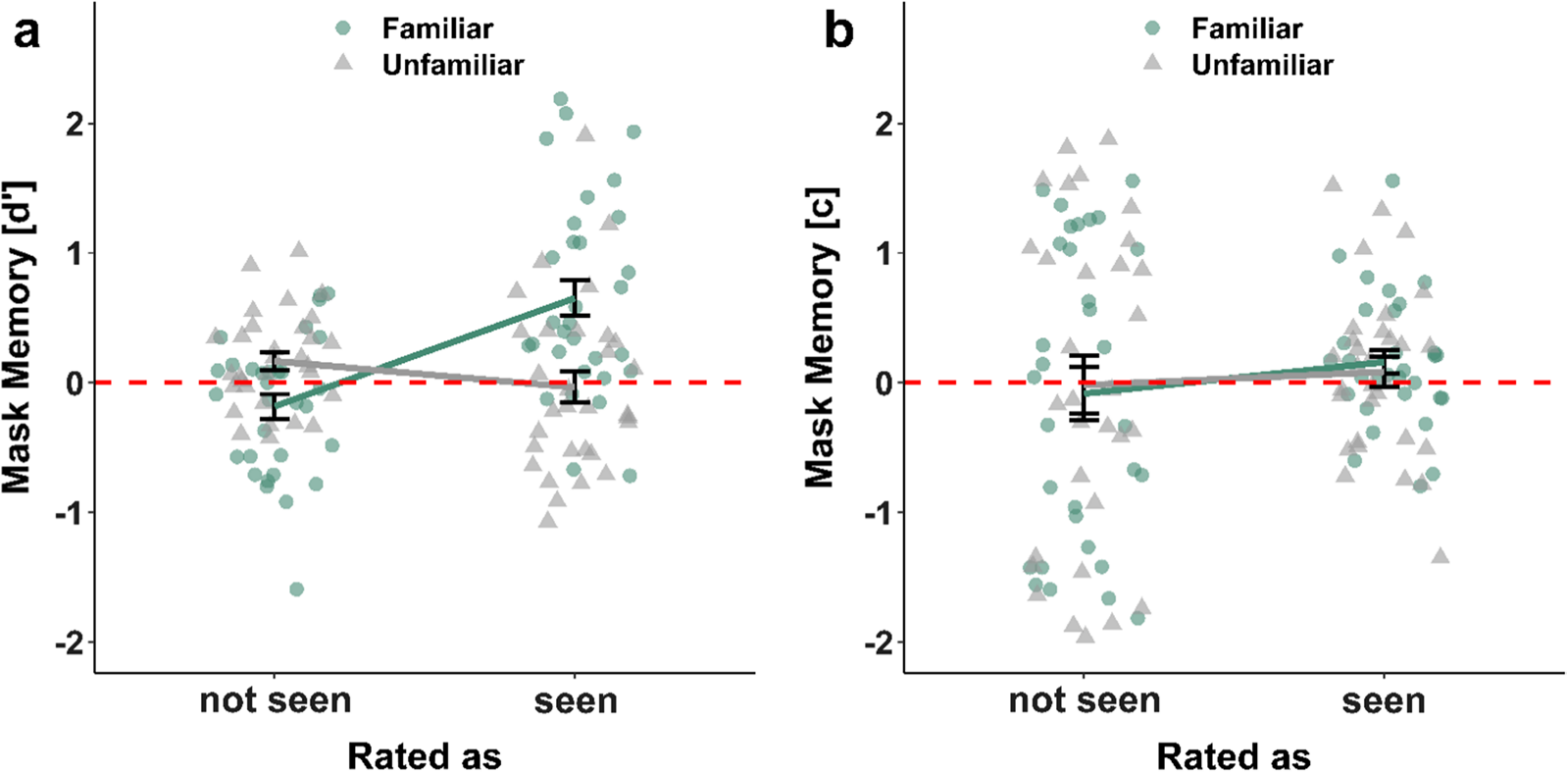
Sensitivity ($d^{\prime }$) and response bias (*c*) for mask memory (Question 2). *Note.* Error bars indicate standard errors. Each data point shows the sensitivity ($d^{\prime }$; panel **a**) or response bias (*c*; panel **b**) of a participant indicating whether they saw a mask in the study phase, separately for cases in which they rated the faces as seen or not seen in the study phase and familiar (blue dots) or unfamiliar faces (gray dots). **a** Main effects of familiarity and rating on memory sensitivity for masks. Higher values of $d^{\prime }$ indicate better performance. A value of zero indicates ‘guessing.’ **b** Main effects of familiarity and rating on memory bias for masks. Values greater than zero indicate a conservative bias (tendency to answer ‘no’), and values lower than zero indicate a liberal bias (tendency to answer ‘yes’)

Participants were significantly better in distinguishing whether a familiar face was wearing a mask (answer to Question 2) compared to unfamiliar ones, when they indicated they saw the face in the study phase (Question 1), *t*(30) = 3.62, *p*_*adj*_ = 0.003, *d* = 0.65. There was a significant effect for a better mask memory for unfamiliar faces compared to familiar ones, when rated as not seen, *t*(30) =  − 3.49, *p*_*adj*_ = 0.003, *d* =  − 0.63. In addition, participants were better in distinguishing whether a familiar face wore a mask, when they rated the face as seen in the study phase compared to when they rated it as not seen, *t*(30) =  − 4.76, *p*_*adj*_ < 0.001, *d* =  − 0.86. There was no difference between ratings for unfamiliar faces, *t*(30) = 1.45, *p*_*adj*_ = 0.158, *d* = 0.26 (Fig. 6a). Additionally, Table 5 summarizes the comparisons of the measure $d^{\prime }$ with zero, depending on the different factor levels. Participants’ mask memory was significantly better than chance only for faces that were *both*, familiar and rated as seen.

**Table 5:** Memory for masks (Question 2)

	*t*	95% CI	*p*_adj_	*d*
*Familiar*				
Rated as seen	4.73	0.37, 0.93	< .001*	0.85
Rated as not seen	− 1.9	− 0.38, 0.01	.133	− 0.34
*Unfamiliar*				
Rated as seen	− 0.28	− 0.28, 0.21	.778	− 0.05
Rated as not seen	2.5	0.03, 0.3	.055	0.45

One sample *t* tests comparing the sensitivity ($d^{\prime }$) with zero

*Marks statistically significant results. Results are shown separately for cases in which participants rated the faces as seen or not seen in the study phase (Question 1)

Note that the results reported here are based on a set criterion of a minimum of 5 trials per cell per participant (cf. Additional file 1: Material S6) to ensure a more balanced comparison between the different conditions. Because of this criterion, we had to exclude 33 participants, jeopardizing statistical power. In Additional file 1: Analysis S7, we report an additional analysis that used a more liberal inclusion criterion of a minimum of 2 trials per cell. Overall, we replicated the results reported here for sensitivity ($d^{\prime }$). However, when we set the criterion to a minimum of 2 trials, there was a significant liberal response bias to indicate that familiar and unfamiliar faces wore a mask when the faces were rated as not seen. We opted to present results for the more conservative inclusion criterion in the main text as an extremely low number of trials per condition per participants risk biases in signal detection analyses.

### Effect of graded familiarity

Most studies investigating the influence of familiarity on face recognition compare unfamiliar, never previously seen, faces to highly familiar people, often well-known celebrities in a binary fashion (as we did in this study). However, familiarity may be a spectrum in the sense that we know some people better than others (Kramer et al., 2018) and remember encounters with them more accurately (e.g., their wearing of a mask), the more familiar those people are. Therefore, we computed post hoc correlation analyses with data from Experiments 1 and 4 and could observe that familiarity ratings of the 200 identities correlate positively with the sensitivity ($d^{\prime }$) to remember those faces with masks, *t*(198) = 5.04; *r* = 0.34; *p* < 0.001, and those without masks, *t*(198) = 6.9; *r* = 0.44; *p* < 0.001. In addition, the familiarity ratings of familiar and unfamiliar identities correlate positively with the sensitivity to remember masks, *t*(98) = 5.58; *r* = 0.49; *p* < 0.001. Note that in the Additional file 1: Analysis S4, we reported an additional post hoc analysis of the data from Experiment 3 that considers familiarity as a continuous variable.

## General discussion

In order to reduce the transmission of COVID-19, many countries introduced non-pharmacological interventions, such as contact tracing and mandatory mask wearing in public areas. In this study, we tested how well participants perform at remembering faces and their wearing of masks as a function of face familiarity. The results can inform contact tracing strategies, which often rely on accurate memory of both, the identity and mask wearing of recent contacts.

### Memory performance for face identity

As expected, our results replicated the finding that participants remember familiar faces better than unfamiliar ones (cf. Johnston & Edmonds, 2009), even when these faces wore a mask during the study phase (cf. Carragher & Hancock, 2020). This is in line with the robust familiar face recognition shown in the literature. However, here we show that face masks have a detrimental effect on recognizing faces, regardless of their familiarity (no significant interaction between familiarity and masks). Previous research found a more detrimental effect of face masks on unfamiliar compared to familiar face matching in cross-experimental comparisons (Noyes et al., 2021). The results by Carragher and Hancock (2020) showed a trend in the opposite direction. However, this effect was interpreted as inconsequential by the authors because of different baseline performances for the two conditions. In addition, their observed effect sizes indicated an almost identical impairment when matching familiar and unfamiliar faces with masks compared to faces without masks.

Our results support a comparable detrimental effect of face masks on familiar and unfamiliar face recognition. Thus, masks could extend Johnston & Edmonds’ list of factors that have detrimental effects on both familiar and unfamiliar face recognition such as lighting, negation and inversion (Johnston & Edmonds, 2009). This would be in line with a recent study by Freud et al. (2020), which showed that holistic face processing (Farah et al., 1998) is impeded by face masks. In addition, a study by Leder and Carbon (2005) demonstrated that when parts of a face were learned in isolation (e.g., the eye region) and later embedded in a full face, recognition accuracy decreased significantly. This suggests that the context of the whole face can interfere with the recognition of its parts. Also, transformations between study (with mask) and test images (without mask) have likely reduced the recognition performance for both, familiar and unfamiliar faces (Patterson & Baddeley, 1977; Terry, 1994). Even minor changes in the appearance of a face can lead to the perception of a change in identity (Graham & Ritchie, 2019). For instance, this ‘incongruency’ effect has been shown with face matching tasks that compared faces with and without wearing ski masks (Manley et al., 2019), glasses (Kramer & Ritchie, 2016) or different eyewear (Graham & Ritchie, 2019). Finally, face masks cover internal facial features such as the nose, mouth and chin. Internal features are believed to be expressive but tend to remain relatively stable over time (e.g., are not disrupted by changes in hair style, etc.), which is why participants tend to rely on them, especially during familiar face recognition (e.g., Ellis et al., 1979). Taken together, the disruption of holistic face processing, image transformations between study and test phase as well as the coverage of internal face features likely explain the detrimental effect on both familiar and unfamiliar face recognition. However, as Noyes et al. (2021) found a difference between these conditions, more research is needed to clarify the potential interaction between familiarity and masks on face recognition.

Although participants showed a stronger conservative bias (tendency to answer that a face was not seen) for unfamiliar faces that wore a mask (cf. Noyes et al., 2021), they showed this conservative bias regardless of whether a face had worn a mask or not in the study phase. This is in contrast to the results regarding unfamiliar faces from Carragher and Hancock’s (2020) study. In the unmasked condition of their Glasgow face matching test, participants showed no response bias, whereas the participants in the mixed condition (one of two faces was shown with a mask) showed a conservative response bias (they tended to perceive a mismatch). In addition, using the Stirling famous face matching task, the authors observed no response bias for unfamiliar faces in the unmasked as well as mixed conditions. One explanation for these discrepant results could be that the authors used a between design concerning their mask conditions, which may have reduced statistical power. Another reason may be that Carragher and Hancock (2020) used a matching task, which likely targets processes different from a memory task (cf. Fysh, 2018). Whereas face matching tasks require participants to recognize whether two pictures show the same identity, the test phase in face memory tasks requires a comparison of the face being tested with stored representations of faces that have occurred in a previous study phase. This difference may also become evident, when comparing the liberal bias in matching familiar faces (tendency to perceive a match) that wore a mask in Carragher and Hancock's (2020) study and our results, which did not show a significant bias for familiar faces. In summary, our results suggest a bias not to remember unfamiliar faces with and without masks, whereas no bias was evident for familiar faces.

A practical implication of these results is that contact tracers should be aware of the tighter memory bottleneck for unfamiliar faces. They could emphasize situations in which the infected person may have encountered unfamiliar people (e.g., public events, restaurants) in interviews and, if possible, try to use additional resources such as guest lists to infer those present at the same time.

### Memory performance for mask wearing

As we and others (for a review, see Johnston & Edmonds, 2009) found that memory for familiar faces is better compared to unfamiliar faces, one might expect that other aspects of encoding are superior for familiar faces too. Indeed, in Experiment 3, we found that participants had better memory for face masks worn by familiar faces compared to unfamiliar faces. They were more biased to indicate that unfamiliar faces wore a mask. This bias may reflect an implicit strategy in which participants are more likely to indicate that they saw a face wearing a mask when they are uncertain about having seen the face at all. Participants may attribute their poor memory for (mostly) unfamiliar faces to them wearing masks, regardless of whether they actually wore a mask.

In Experiment 4, we asked participants to indicate which faces they remembered and subsequently based our analysis of a second question, regarding mask memory, on these ratings. This implied a reduction in statistical power (see below, limitations), which we bear in mind when interpreting the effects on mask memory observed in Experiment 4. We cautiously conclude from these results that both familiarity and identity memory are important factors for remembering whether a given face wore a mask. Participants memory for masks was only above chance level when they indicated remembering the face *and* the face was familiar. Hence, memory for mask wearing in contact tracing situations might be compromised in situations where unfamiliar people gather (e.g., public transport), but more accurate for settings in which familiar people interact (e.g., classroom, sports club).

The practical implications of these findings have to be considered in light of the fact our observers had to remember face ‘encounters’ on a screen rather than real-life interactions. Further research with higher ecological validity is needed for certain conclusions. Nevertheless, our findings suggest that restricting contact tracing interviews to ‘relevant’ encounters and defining them by mask wearing risks a high number of avoidable misses, especially for settings in which interviewees interacted with unfamiliar people. Health authorities and contact tracers may consider dropping such restrictions, at least for encounters with unfamiliar people.

## Limitations

Although the current study is a first approach to better understand the effect of familiarity on memory for faces and mask wearing, there are important limitations and open questions which should be addressed in future studies.

First, our analysis of Experiment 4 grouped mask memory responses depending on face recognition (seen/unseen). This led to unbalanced trial numbers for the eight resulting cells crossing seen and unseen with familiar and unfamiliar as well as masked and unmasked faces. Critically, for many participants this trial number was below a minimum of five trials for at least one of the cells (Additional file 1: Material S6) and they had to be excluded from this analysis. This left us with reduced power and cautious implications concerning the effect of familiarity and face recognition on mask memory. This power reduction may explain why we could not replicate our finding from Experiment 3, demonstrating that participants were biased to indicate that unfamiliar faces wore a mask. We reported an additional analysis with a more liberal inclusion criterion of a minimum of two trials in Additional file 1: Analysis S7. Although these results should be taken with caution due to the very low number of trials per condition for some participants and resulting biases in signal detection analyses, they suggest that faces reported as unseen in the study phase are more likely to be assigned a mask, regardless of familiarity.

Furthermore, our current experiments tested memory for faces and masks in a highly artificial, online setting and over very short periods of time (the test phase followed the study phase immediately). Contact tracing relies on memory for identities and their wearing of masks for up to 14 days before diagnosis (World Health Organization, 2021b), which presumably is a lot harder. Future experiments should systematically study the effects of different delays between study and test phase. At the same time, real-life situations provide many additional cues compared to our single image trials, which may significantly *improve* recognition performance, especially for occluded faces. For example, the dynamics of face and body motion may provide additional information about a familiar facial identity (Lander et al., 1999; Pike et al., 1997) and its idiosyncratic variability (e.g., different viewpoints and expressions) (cf. Bruce, 1982; Jenkins et al., 2011; Young & Burton, 2017). In this sense, one might ask how far encountering faces with and without face masks in photographs is truly representative of natural encounters. However, in a study by Bruce et al. (1999), watching short video clips of unfamiliar identities in comparison with static images increased the recognition performance only slightly. Consequently, it has been argued that the recognition of static images is already so good that additional idiosyncratic patterns of facial movement convey no meaningful benefit under normal circumstances. Only when significant image degradation occurs, positive effects of facial movements become apparent (Lander et al., 1999; O’Toole et al., 2002). In addition, a study using videos indicated that obscuring the heads had a dramatic effect on participants’ ability to recognize an identity, while obscuring the body or gait produced only a small decrement in recognition performance (Burton et al., 1999). Future research should address this question emulating real-life situations and/or varying information content (e.g., whole body vs. face only, movement and wearing of face masks).

In our study design, half of the faces in the study phase were shown with a mask, while all faces in the test phases of Experiments 3 and 4 were shown without a mask. Thus, encoding and retrieval were matched in the (congruent) unmasked but not in the (incongruent) masked condition, which is associated with a decrease in memory performance (cf. Marini et al., 2021; see also Bobak et al., 2019). Following the logic that memory performance is better in congruent trials, one might expect a better performance in a congruent condition where faces in the study and test phase wear masks compared to the incongruent condition. However, Carragher and Hancock (2020) reported that matching performance was impaired to the same degree for trials in which one or both faces were covered with a mask (Carragher & Hancock, 2020). These results could also apply to memory performance, but this would need to be empirically tested in the future. Nevertheless, in terms of contact tracing, it is probably more ecologically valid to show unconcealed faces in the test phase. For example, one could consider a scenario in which individuals are asked about their un-/protected contacts at a past event and search social media profiles to aid their memory. In most cases, the profile pictures will show people without masks.

Here, we used different samples for assessing the familiarity of faces and the memory tasks. The reported positive correlations of graded familiarity could be even stronger when probing familiarity on an individual basis in the same participants completing the memory tests. However, by explicitly choosing familiar and unfamiliar faces from a larger pool of celebrities, the design of the study was intended to make a categorical distinction between familiarity, akin to extreme group approaches in individual differences research, which have been shown to maximize power (cf. de Haas, 2018; Preacher, 2015). Additionally, one may argue that aiming for a bimodal distribution of familiarity is ecologically valid for the context of daily encounters potentially relevant for contact tracing. Contact tracing interviews typically move from ‘inner circles’ in which contacts are highly familiar (co-habitants, colleagues, etc.) to outer circles in which contacts are typically entirely unfamiliar (public transport, shopping, etc.). Nevertheless, future studies may choose their stimuli in order to reflect a whole spectrum of familiarity and obtain post hoc familiarity ratings from all participants to test the effects of graded familiarity in a more fine-grained, sensitive manner. Moreover, the ability to recognize faces and distinguish between them differs largely between individuals, with extreme manifestations ranging from ‘Super-recognizers’ (SRs, very good face recognition skills; e.g., Russell et al., 2009) to ‘Developmental Prosopagnosics’ (very poor face recognition skills; e.g., Duchaine & Nakayama, 2006). A study by Noyes et al. (2021) investigated the effect of face masks on SRs’ and control participants’ matching performance and demonstrated that both were equally impaired by occlusion. However, at the group level, they outperformed control participants. Moreover, Bennetts et al. (2022) observed that control participants had a tendency to respond more conservatively than SRs, when one of two face images wore a mask. In line with Noyes et al. (2021), they also observed that both control participants’ and SRs’ matching performance decreased when one face was wearing a mask. However, there was no significant difference between their matching performances. In line with the observations from SRs, self-reported recognition ability within the typical population showed a small but negative correlation with the magnitude of mask effects on accuracy in matched identity trials (but not other measures of performance). Interestingly, extended natural exposure of adults to faces wearing masks during the COVID-19 pandemic did not lead to an improvement in recognizing masked faces (Bennetts et al., 2022; Freud et al., 2021). In the present study, we did not investigate to what extent general face recognition ability or the amount of exposure to masks correlates with mask memory and this could be an interesting avenue for future research.

## Conclusion

In this study, we tested how memory for faces and their wearing of masks interact and are influenced by familiarity. In four online studies, we validated a stimulus set containing identities that were familiar or unfamiliar to our test population (Experiment 1) and found a memory advantage for the identity of familiar (Experiments 2 and 4) and unmasked faces, with no interaction between these factors (Experiment 4). We also found that the ability to remember whether a face wore a mask is better for familiar faces (Experiment 3) whose identity was remembered (Experiment 4). Furthermore, participants showed a significant conservative bias to indicate no memory of unfamiliar faces and a significant liberal bias to indicate that unfamiliar faces wore a mask (Experiment 3). We conclude that it is difficult to accurately remember faces and their wearing of masks, especially for short contacts with strangers

## Supplementary Information

**Additional file 1.** Additional information on the instructions and images shown in the experiments and additional control analyses for Experiment 2, 3, and 4.

## Data Availability

The stimuli used as well as datasets generated and analyzed during the current study are available for download on the Open Science Framework [https://osf.io/fcxaj/]. A preprint of this work is maintained on PsyArXiv [https://psyarxiv.com/tm54k/].

## Abbreviations

CFP: Celebrities in Frontal-Profile (refers to the dataset we used)
*d*′: ‘D-prime’ = Sensitivity
*c*: Criterion = Response bias
SDT: Signal detection theory
SR: Super-recognizer
