# Supplementary material for: The influence of familiarity on memory for faces and mask wearing
Source: Cogn Res Princ Implic. 2022 May 15;7:45. doi: 10.1186/s41235-022-00396-4 (PMC9107586; doi:10.1186/s41235-022-00396-4)
Supplement: Supplementary file 1 — Additional file 1. Additional information on the instructions and images shown in the experiments and additional control analyses for Experiment 2, 3, and 4. [file 41235_2022_396_MOESM1_ESM.docx]

**Additional file 1**

**The Influence of Familiarity on Memory for Faces and Mask Wearing**

Diana Kollenda & Benjamin de Haas

**Additional file 1: Text S1**

Instructions (original in German):

Experiment 1

„Im folgenden Experiment werden Sie eine Reihe von Gesichtern sehen - und wir möchten wissen, ob Sie das jeweilige Gesicht kennen, d.h. schon mal (vor dem Experiment!) gesehen haben und ggf. den Namen der Person erinnern.

Bitte wählen Sie dafür eine der folgenden drei Optionen mit den entsprechenden Zahlen auf Ihrer Tastatur:

(1) Nein.

(2) Ja, kenne ich, aber ich weiß nicht mehr woher.

(3) Ja, kenne ich (ggf. namentlich).

Bitte drücken Sie auf "Weiter", um mit dem Experiment zu beginnen.“

Experiment 2, 3 & 4

Study phase:

Im folgenden Experiment werden Sie eine Reihe von Gesichtern sehen - und wir möchten wissen, welchem Geschlecht Sie dem jeweiligen Gesicht zuordnen.

Bitte wählen Sie dafür eine der folgenden drei Optionen mit den entsprechenden Zahlen auf Ihrer Tastatur:

(1) Mann

(2) Frau

(3) Divers

Bitte drücken Sie auf "Weiter", um mit dem Experiment zu beginnen.“

Experiment 2

Test phase:

Im folgenden Teil des Experiments werden Sie eine Reihe von Gesichtern sehen - und wir möchten wissen, ob Sie das jeweilige Gesicht in dem vorherigen Durchgang des Experiments gesehen haben.

Bitte wählen Sie dafür eine der folgenden drei Optionen mit den entsprechenden Zahlen auf Ihrer Tastatur:

(1) Nein, ich habe dieses Gesicht im ersten Durchgang des Experiments nicht gesehen.

(2) Eher nein.

(3) Eher ja.

(4) Ja, ich habe dieses Gesicht im ersten Durchgang des Experiments gesehen.

Bitte drücken Sie auf "Weiter", um mit dem Experiment zu beginnen.“

Experiment 3

Test phase:

„Im folgenden Teil des Experiments werden Sie eine Reihe von Gesichtern sehen - und wir möchten wissen, ob Sie glauben, dass Sie das jeweilige Gesicht in dem vorherigen Durchgang des Experiments mit oder ohne Maske gesehen haben.

Bitte wählen Sie dafür eine der folgenden zwei Optionen mit den entsprechenden Zahlen auf Ihrer Tastatur:

(1) Nein, ich habe dieses Gesicht im ersten Durchgang des Experiments ohne Maske gesehen.

(2) Eher nein.

(3) Eher ja.

(4) Ja, ich habe dieses Gesicht im ersten Durchgang des Experiments mit Maske gesehen.

Bitte drücken Sie auf "Weiter", um mit dem Experiment zu beginnen.“

Experiment 4

Test phase:

Im folgenden Teil des Experiments werden Sie eine Reihe von Gesichtern sehen - und wir werden Ihnen zwei Fragen zu jedem dieser Gesichter stellen.

1. Frage: Wir möchten wissen, ob Sie glauben, dass Sie das jeweilige Gesicht in dem vorherigen Durchgang des Experiments gesehen haben (unabhängig davon, ob Sie es mit oder ohne Maske gesehen haben).

Bitte wählen Sie dafür eine der folgenden Optionen mit den entsprechenden Zahlen auf Ihrer Tastatur:

(1) Nein, ich habe dieses Gesicht im ersten Durchgang des Experiments nicht gesehen.

(2) Eher nein.

(3) Eher ja.

(3) Ja, ich habe dieses Gesicht im ersten Durchgang des Experiments gesehen.

2. Frage: Angenommen, dieses Gesicht wurde im ersten Teil des Experimentes dargeboten. Wurde es mit oder ohne Maske gezeigt?

Bitte wählen Sie dafür eine der folgenden Optionen mit den entsprechenden Zahlen auf Ihrer Tastatur:

(1) Ich habe das Gesicht ohne Maske gesehen.

(2) Eher ohne Maske.

(3) Eher mit Maske.

(4) Ich habe das Gesicht mit Maske gesehen.

Bitte drücken Sie auf "Weiter", um mit dem Experiment zu beginnen.“

**Additional file 1: Stimulus Figures S2**

**Figure S2.1**

*Familiar image set (two frontal images per identity)*


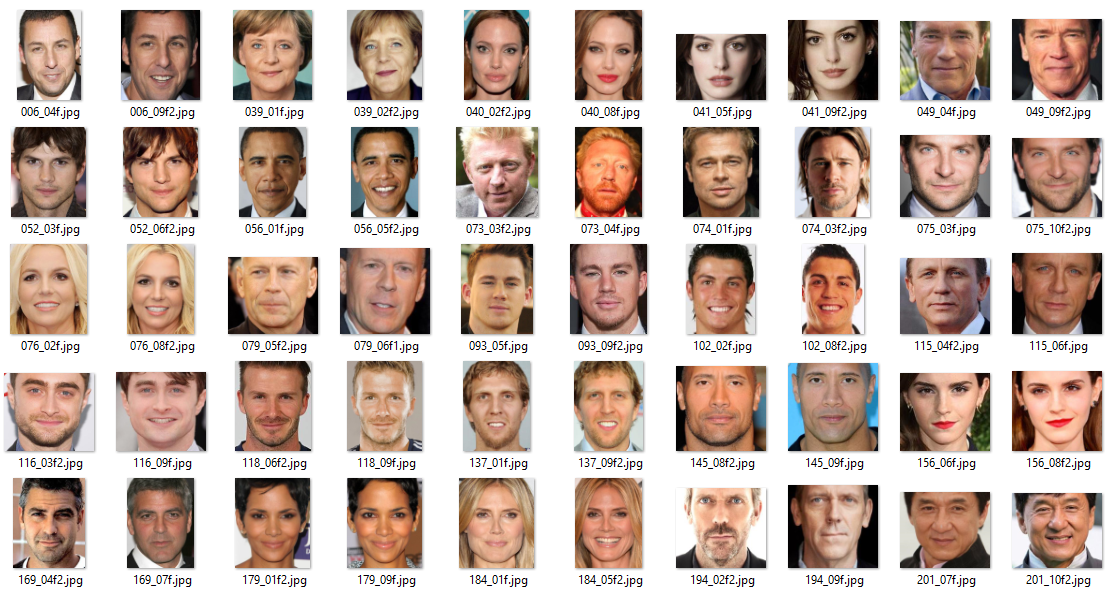

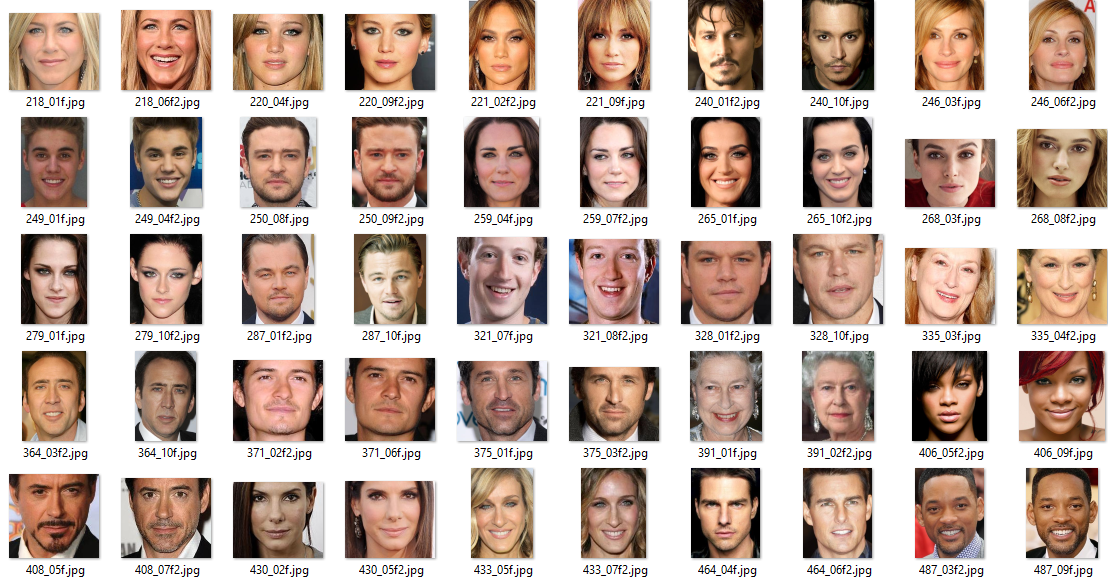


**Figure S2.2**

*Familiar faces with mask*


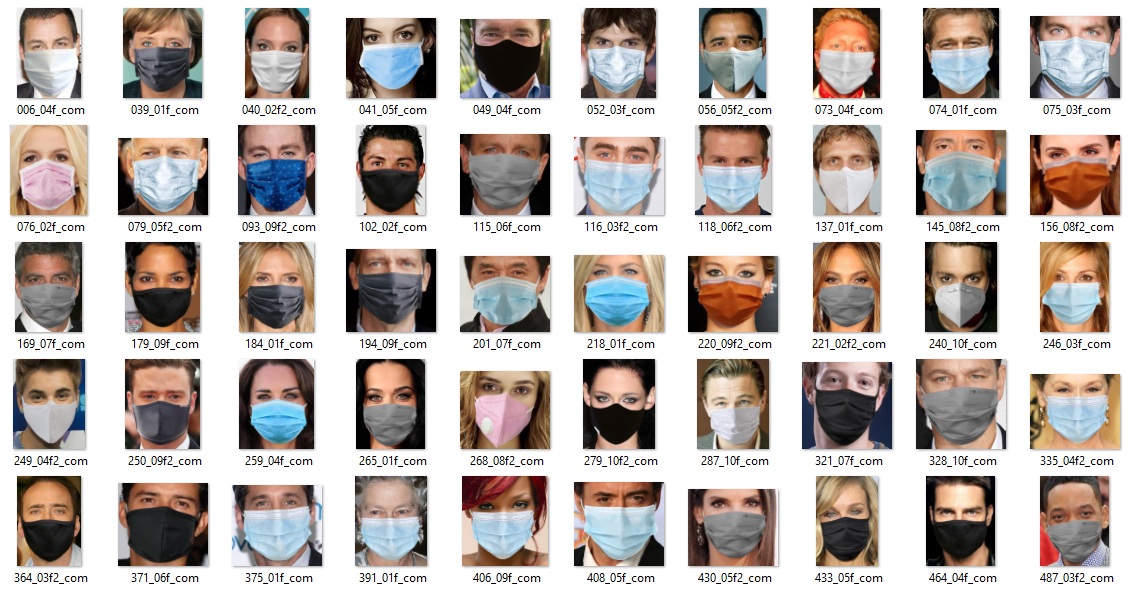


**Figure S2.3**

*Unfamiliar image set (two frontal images per identity)*


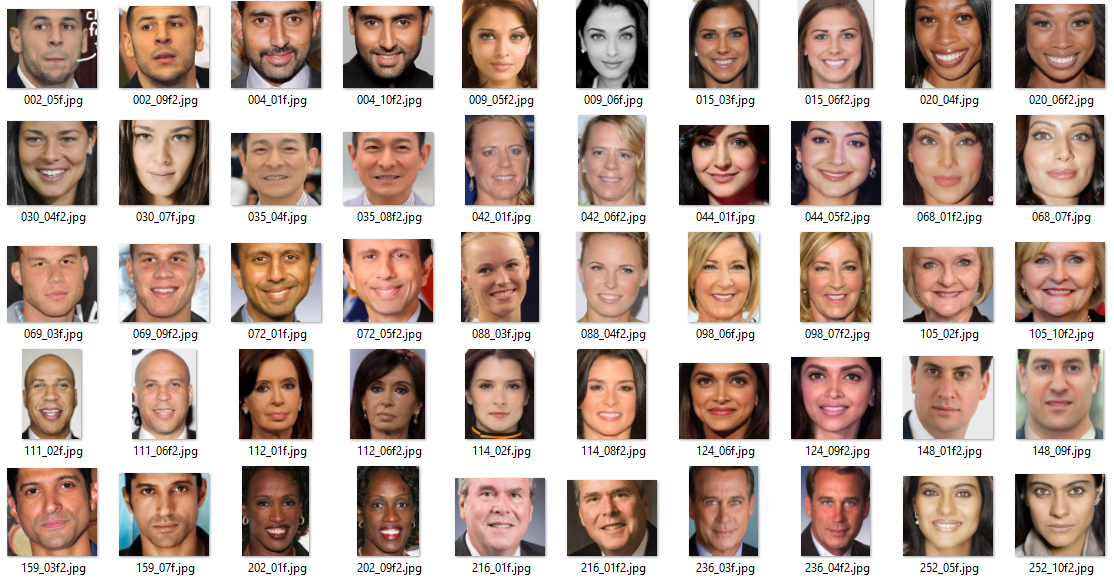

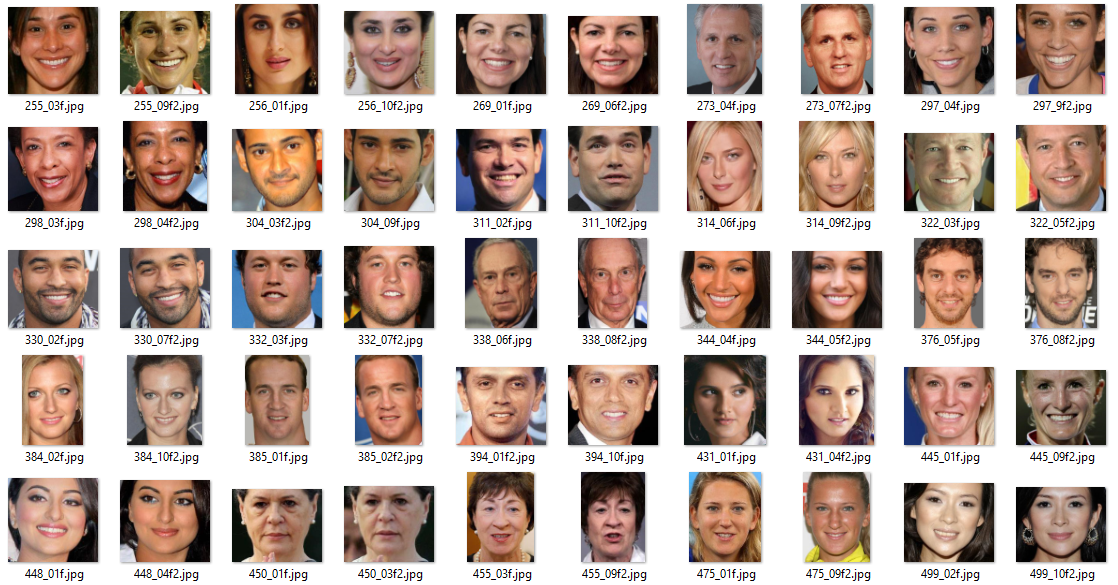


**Figure S2.4**

*Unfamiliar faces with mask*


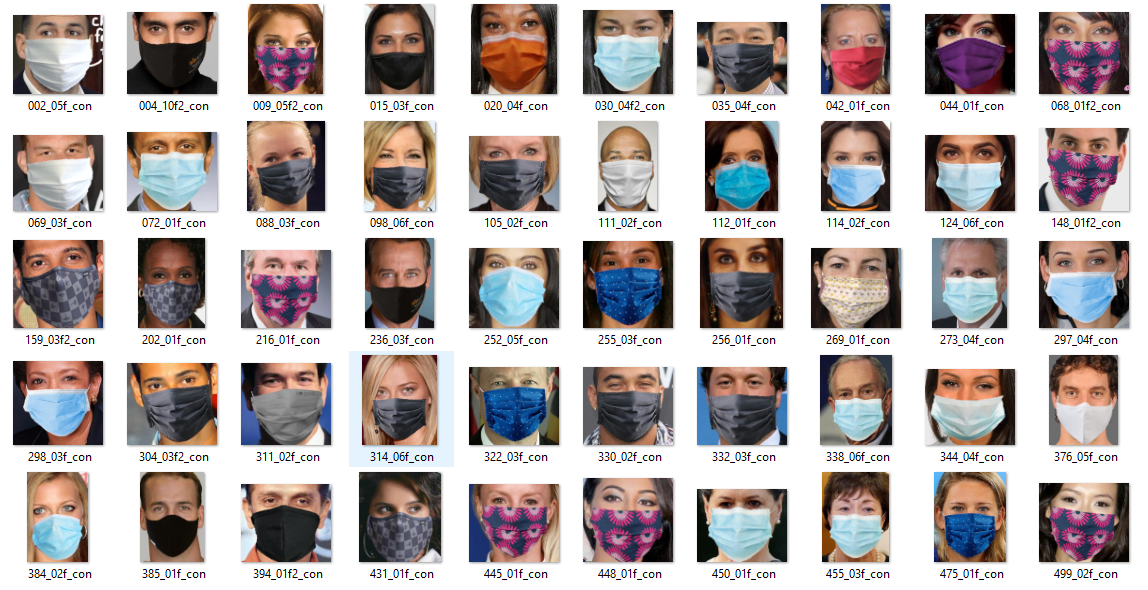


**Figure S2.5**

*Lure_familiar image set*
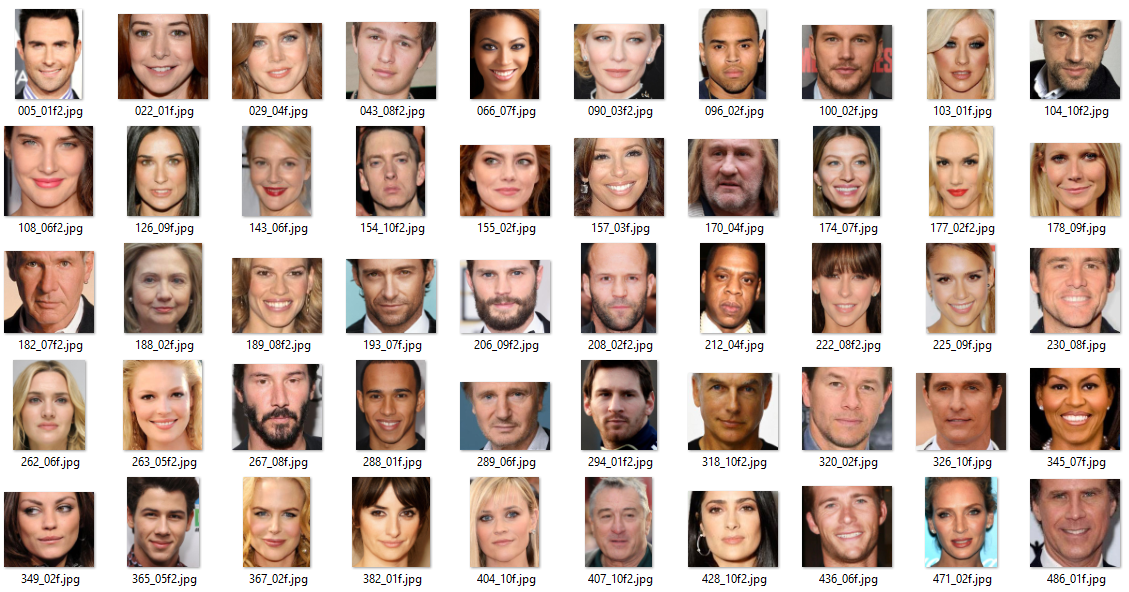


**Figure S2.6**

*Lure_unfamiliar image set*
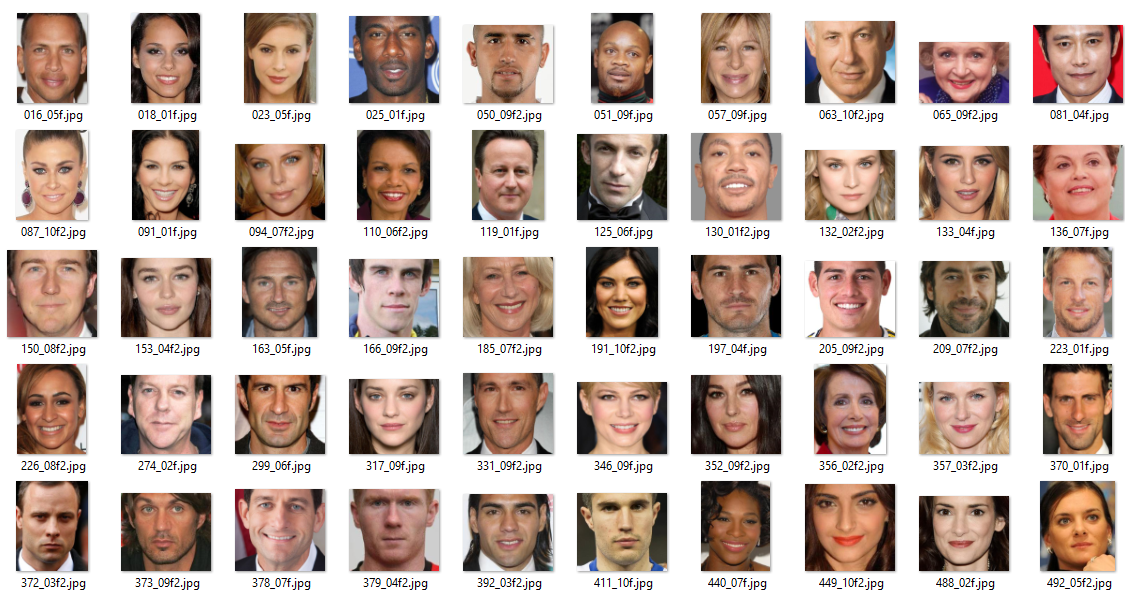


**Additional file 1: Control Analysis S3**

Due to the way we designed our target and lure stimuli, participants may have profited from a ‘familiarity heuristic’ in the familiar condition (i.e., if participants chose the more familiar face it would lead to better performance), which at the same time would have been detrimental in the unfamiliar condition. Here we tested whether the observed familiarity advantage in Experiment 2 was robust against subtracting the estimated size of this confounding factor (‘corrected data’, see Methods for details). First, we regressed the frequency of ‘yes’ responses (likely seen or seen) onto familiarity across all lure trials, which yielded an estimated beta of 2.73 for the familiarity heuristic per point on the familiarity scale and across 100 trials. We then multiplied this number with the average familiarity difference between familiar lures and targets (Diff_familiar_ = 0.59) and adjusted it for the lower number of trials in each cell (50, i.e. factor 0.5) to estimate the additional hits and lacking false alarms in the familiar condition due to this confound(0.5(beta * Diff_familiar_) = 0.81). This allowed us to estimate a downward ‘corrected’ d' for the familiar condition by converting the respective number of hits to misses and correct rejections to false alarms for each individual. Similarly, we multiplied the estimated effect size of the familiarity heuristic with the average familiarity difference between unfamiliar lures and unfamiliar targets (Diff_unfamiliar_ = 0.25) to convert the respective number (0.5(beta * Diff_unfamiliar_) = 0.34) of misses to hits and false alarms to correct rejections, thus upward ‘correcting’ the d’ for the unfamiliar condition. Finally, we tested the robustness of the familiarity advantage, by comparing the corrected d’ between conditions.

Descriptive statistics for d’ and c are reported in Table S3.1 In total, participants had a corrected mean (SD) hit rate of 33.52 (8.0) for familiar faces and 22.2 (7.35) for unfamiliar faces. They had a corrected mean (SD) false alarm rate of 14.62 (8.34) for familiar face lures and 12.17 (7.75) for unfamiliar face lures.

**Table S3.1**

*Memory for Face Identities, Depending on the Familiarity of Faces. Descriptive Statistics [mean(standard error of the mean)] for the Measures Sensitivity (d’) and Criterion (c)*

|  | Sensitivity (d‘) | | Response Bias (c) | |  |  |
| --- | --- | --- | --- | --- | --- | --- |
|  | Familiar | Unfamiliar | Familiar | Unfamiliar | | |
| Uncorrected | 1.17(0.09) | 0.57(0.07) | 0.06(0.06) | 0.45(0.06) | | |
| Corrected | 1.06(0.09) | 0.62(0.07) | 0.06(0.06) | 0.45(0.06) | | |

*Note.* Results are shown separately for uncorrected and corrected data.

**Table S3.2**

*Memory for Face Identities, Depending on the Familiarity of Faces. One Sample* t *Tests Comparing the Sensitivity (d’) and Response Bias (c) with Zero*

|  | *t* | *95% CI* | *p_adj_* | *d* | |
| --- | --- | --- | --- | --- | --- |
| Sensitivity (d’) |  |  |  |  | |
|  | Familiar |  |  |  | |
| Uncorrected | 12.74 | 0.98, 1.36 | <.001* | | 1.94 |
| Corrected | 11.88 | 0.88, 1.24 | <.001* | | 1.81 |
|  | Unfamiliar |  |  | |  |
| Uncorrected | 8.57 | 0.44, 0.7 | <.001* | | 1.31 |
| Corrected | 9.1 | 0.48, 0.75 | <.001* | | 1.39 |
|  |  |  |  | |  |
| Response Bias |  |  |  | |  |
|  | Familiar |  |  | |  |
| Uncorrected | 0.98 | -0.06, 0.18 | .33 | | 0.15 |
| Corrected | 0.97 | -0.06, 0.17 | .336 | | 0.15 |
|  | Unfamiliar |  |  | |  |
| Uncorrected | 7.27 | 0.32, 0.57 | <.001* | | 1.11 |
| Corrected | 7.25 | 0.33, 0.58 | <.001* | | 1.11 |
|  |  |  |  | |  |

*Note.* * marks statistically significant results. Results are shown separately for uncorrected and corrected data.

**Table S3.3**

*Memory for Face Identities.* t *Tests Comparing the Sensitivity (d’) and Response Bias (c) of Familiar and Unfamiliar faces*

|  | *t* | *95% CI* | *p_adj_* | *d* | |
| --- | --- | --- | --- | --- | --- |
| Sensitivity (d’) |  |  |  |  | |
| Uncorrected | 9.57 | 0.47, 0.73 | <.001* | | 1.46 |
| Corrected | 7.28 | 0.32, 0.57 | <.001* | | 1.11 |
|  |  |  |  | |  |
| Response Bias |  |  |  | |  |
| Uncorrected | -7.54 | -0.49, -0.29 | <.001* | | -1.15 |
| Corrected | -7.7 | -0.5, -0.29 | <.001* | | -1.17 |
|  |  |  |  | |  |

*Note.* * marks statistically significant results. Results are shown separately for uncorrected and corrected data.

**Figure S3**

*Sensitivity (d’) and Response Bias (c) for Identity Recognition in Corrected Data, Depending on the Familiarity of Faces*


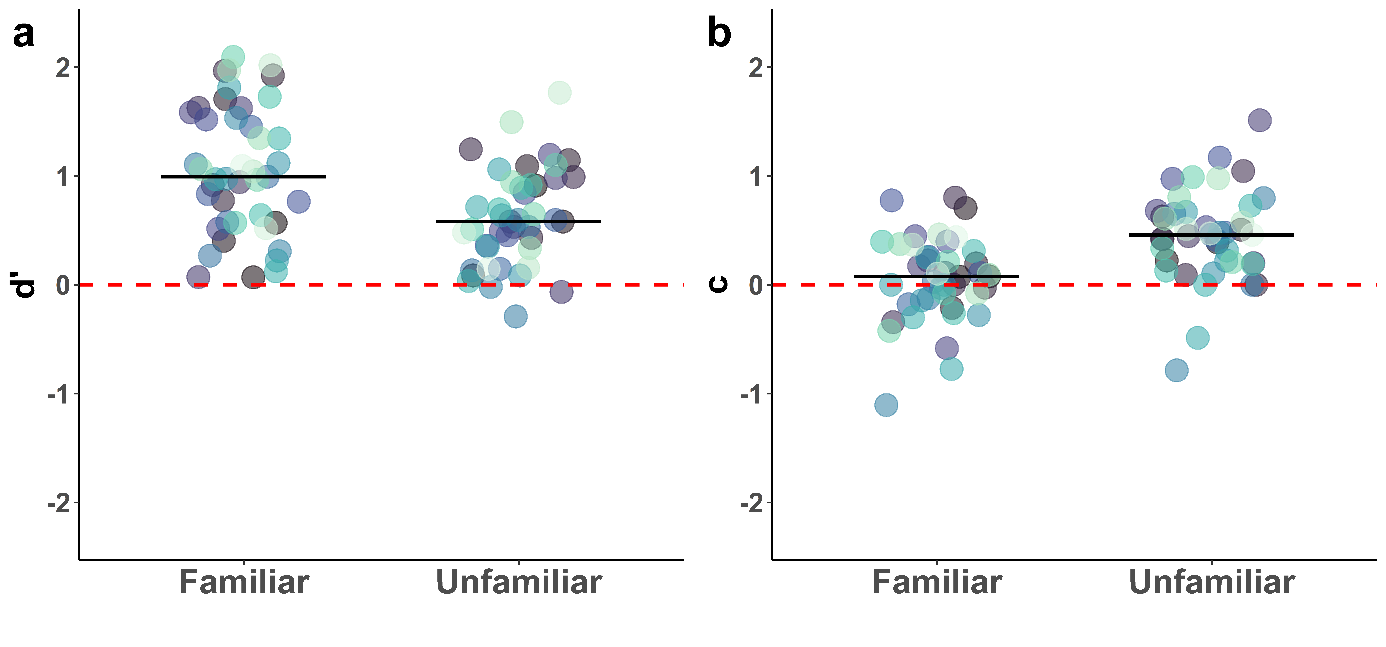


Note. Black horizontal lines indicate median values. Same colors indicate data points from the same participant. Each datapoint shows the sensitivity (d’; panel a) or response bias (c; panel b) of a single participant indicating whether they saw a given face in the study phase, separately for familiar and unfamiliar faces.

a) Advantage for remembering familiar faces. Higher values of d’ indicate better performance. A value of zero indicates ‘guessing’.

b) Stronger conservative bias for unfamiliar faces. Values greater than zero indicate a conservative bias (tendency to answer ‘no’) and values lower than zero indicate a liberal bias (tendency to answer ‘yes’).

Overall, these results are consistent with those reported in the section on the results of Experiment 2. Participants showed a significant memory advantage (higher sensitivity) for familiar faces compared to unfamiliar faces (cf. Table S3.3; Figure S3a). Participants also showed a significantly stronger conservative bias (tendency to indicate no memory) for unfamiliar than familiar faces (cf. Table S3.3; Figure S3b).

**Additional file 1: Analysis S4**

**Figure S4**

*Logistic Mixed-Effect Model with Familiarity and Mask Condition as Fixed Effects*


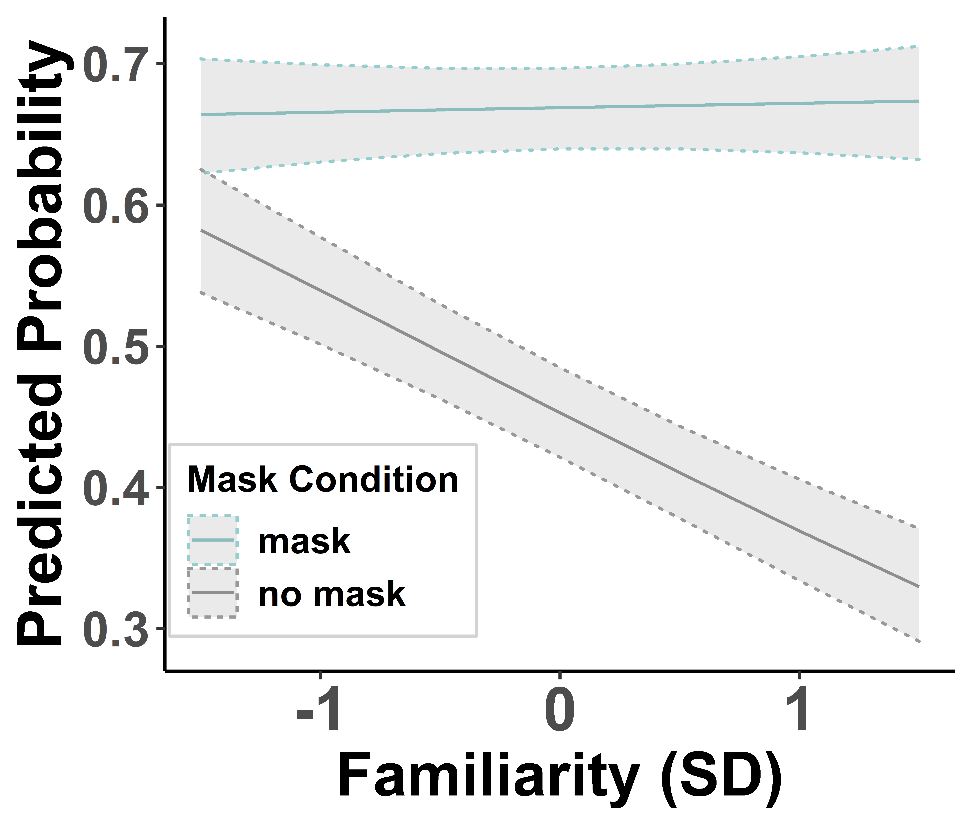


*Note*. Grey shadings represent 95% confident intervals. This plot illustrated the predicted probability of responding with 1 or 2 (i.e., likely yes or yes) that a face was wearing a mask in the study phase of Experiment 3. There is an interaction between the two factors familiarity and mask condition.

Based on data from Experiment 3, we performed a logistic mixed-effects model with random intercepts for each face and participant to assess whether familiarity and mask condition influenced the likelihood of a participant responding with 1 or 2 (i.e. likely yes or yes) instead of -1 or -2 (i.e. likely not or no) to the question whether faces wore a mask in the study phase.

We found no significant effect of familiarity, |𝓏| < 1. The effect of mask condition was significant, β = -0.89, SE = 0.04, 𝓏 = -22.79, p <.001. The interaction between familiarity and mask condition was also significant, β = -0.36, SE = 0.04, 𝓏 = -9.26, p <.001. These results are in line with our results reported for Experiment 3 as both show poorer mask/no mask discrimination for unfamiliar faces, which is driven by a liberal bias for unfamiliar faces. However, in our study design, familiarity was designed as a categorical variable rather than a continuous variable and the analysis reported here should accordingly be interpreted with caution.

**Additional file 1: S5**

Here we tested whether the observed familiarity advantage observed for answers to Question 1 (“Did you see the face in the first part of the experiment?”) in Experiment 4 was robust against subtracting the estimated size of ‘familiarity heuristic’ as confounding factor (‘corrected data’, see Methods for details and cf. Additional file 1: Control Analysis S3). First, we regressed the frequency of ‘yes’ responses (likely seen or seen) onto familiarity across all lure trials, which yielded an estimated beta of 3.66 for the familiarity heuristic per point on the familiarity scale and 100 trials. We then multiplied this number with the average familiarity difference between familiar lures and targets (Diff_familiar_ = 0.59) and a factor adjusting for the lower number of trials per cell (25, i.e. factor 0.25) to estimate the additional hits and lacking false alarms in the familiar condition due to this confound (0.25(beta * Diff_familiar_) = 0.54). This allowed us to estimate a downward ‘corrected’ d' for the familiar condition by converting the respective number of hits to misses and correct rejections to false alarms for each individual. Similarly, we multiplied the estimated effect size of the familiarity heuristic with the average familiarity difference between unfamiliar lures and unfamiliar targets (Diff_unfamiliar_ = 0.25) to convert the respective number (0.25(beta * Diff_unfamiliar_) = 0.23) of misses to hits and false alarms to correct rejections, thus upward ‘correcting’ the d’ for the unfamiliar condition. Note that for two participants, hit and false alarm rates at ceiling and floor were not upward corrected or downward corrected. Finally, we tested the robustness of the familiarity advantage, by comparing the corrected d’ between conditions.

Descriptive statistics for d’ and c are reported in Table S5.1 When faces wore masks in the study phase, participants had a corrected mean (SD) hit rate of 14.63 (4.66) for familiar faces and 9.2 (5.03) for unfamiliar faces. When they were unmasked, participants had a corrected mean (SD) hit rate of 16.79 (4.78) for familiar faces and 11.16 (4.85) for unfamiliar faces. They had a corrected mean (SD) false alarm rate of 17.26 (9.22) for familiar face lures and 14.57 (9.63) for unfamiliar face lures. We conducted a repeated-measured ANOVA with two factors, familiarity (familiar vs. unfamiliar) and presence of a mask (mask vs. no mask) in the study phase for the measures sensitivity (d’) and response bias (c), respectively. For both measures of d’ and c, the main effects of familiarity and mask condition were significant, while the interaction between both factors was not (see Table S5.2).

**Table S5.1**

*Memory for Face Identities (Question 1), Depending on the Familiarity of Faces. Descriptive Statistics [mean(standard error of the mean)] for the Measures Sensitivity (d’) and Criterion (c)*

|  | Sensitivity (d‘) | | Response Bias (c) | |  |  |
| --- | --- | --- | --- | --- | --- | --- |
|  | Familiar | Unfamiliar | Familiar | Unfamiliar | | |
| Uncorrected |  |  |  |  | | |
| Mask | 0.75(0.07) | 0.21(0.05) | 0.08(0.06) | 0.50(0.07) | | |
| No Mask | 1.02(0.09) | 0.44(0.07) | -0.05(0.06) | 0.39(0.07) | | |
|  |  |  |  |  | | |
| Corrected |  |  |  |  | | |
| Mask | 0.65(0.07) | 0.26(0.05) | 0.1(0.06) | 0.49(0.07) | | |
| No Mask | 0.91(0.09) | 0.48(0.07) | -0.03(0.05) | 0.38(0.07) | | |

*Note.* Results are shown separately for familiarity and the presence of a mask in the study phase. Additionally, results are shown separately for uncorrected and corrected data.

**Table S5.2**

*Memory for Face Identities (Question 1). Repeated Measures ANOVA for Sensitivity (d’) and Response Bias (c)*

|  | Sensitivity (d‘) | Response Bias (c) |  |  |  |
| --- | --- | --- | --- | --- | --- |
| Uncorrected |  |  |  |  |  |
| Familiarity | *F*(1, 63) = 85.39, *p* <.001*, η_p_^2^ = .19 | *F*(1, 63) = 82.98, *p* <.001*, η_p_^2^ = .15 | |  |  |
| Mask Condition | *F*(1, 63) = 39.18, *p* <.001*,η_p_^2^ = .04 | *F*(1, 63) = 39.18, *p* <.001*, η_p_^2^ = .01 | |  |  |
| Interaction | *F*(1, 63) = 0.37, *p* = .547, η_p_^2^ = .00 | *F*(1, 63) = 0.37, *p* = .547, η_p_^2^ = .00 | |  |  |
|  |  |  | |  |  |
| Corrected |  |  | |  |  |
| Familiarity | *F*(1, 63) = 48.37, *p* <.001*, η_p_^2^ = .12 | *F*(1, 63) = 74.04, *p* <.001*, η_p_^2^ = .14 | |  |  |
| Mask Condition | *F*(1, 63) = 39.93, *p* <.001*,η_p_^2^ = .04 | *F*(1, 63) = 39.93, *p* <.001*, η_p_^2^ = .01 | |  |  |
| Interaction | *F*(1, 63) = 0.24, *p* = .624, η_p_^2^ = .00 | *F*(1, 63) = 0.24, *p* = .624, η_p_^2^ = .00 | |  |  |

*Note.* * marks statistically significant results. Results are shown separately for uncorrected and corrected data.

**Table S5.3**

*Memory for Face Identities (Question 1), Depending on the Familiarity of Faces. One Sample* t *Tests Comparing the Sensitivity (d’) and Response Bias (c) with Zero*

|  | *T* | *95% CI* | *p_adj_* | *d* | |
| --- | --- | --- | --- | --- | --- |
| Sensitivity (d’) |  |  |  |  | |
|  | Familiar |  |  |  | |
| Uncorrected | 11.3 | 0.73, 1.04 | <.001* | | 1.41 |
| Corrected | 10.1 | 0.63, 0.94 | <.001* | | 1.26 |
|  | Unfamiliar |  |  | |  |
| Uncorrected | 6.12 | 0.22, 0.43 | <.001* | | 0.77 |
| Corrected | 6.92 | 0.26, 0.47 | <.001* | | 0.87 |
|  |  |  |  | |  |
| Response Bias |  |  |  | |  |
|  | Familiar |  |  | |  |
| Uncorrected | 0.28 | -0.1, 0.13 | .783 | | 0.03 |
| Corrected | 0.61 | -0.08, 0.14 | .542 | | 0.08 |
|  | Unfamiliar |  |  | |  |
| Uncorrected | 6.49 | 0.31, 0.58 | <.001* | | 0.81 |
| Corrected | 6.4 | 0.3, 0.57 | <.001* | | 0.8 |

*Note.* * marks statistically significant results. Results are shown separately for uncorrected and corrected data.

**Table S5.4**

*Memory for Masked and Unmasked Faces (Question 1). One Sample* t *Tests Comparing the Sensitivity (d’) and Response Bias (c) with Zero*

|  | *t* | *95% CI* | *p_adj_* | *d* | |
| --- | --- | --- | --- | --- | --- |
| Sensitivity (d’) |  |  |  |  | |
|  | Mask |  |  |  | |
| Uncorrected | 8.73 | 0.37, 0.59 | <.001* | | 1.09 |
| Corrected | 8.25 | 0.34, 0.57 | <.001* | | 1.03 |
|  | No mask |  |  | |  |
| Uncorrected | 10.47 | 0.59, 0.87 | <.001* | | 1.31 |
| Corrected | 10.12 | 0.56, 0.83 | <.001* | | 1.27 |
|  |  |  |  | |  |
| Response Bias |  |  |  | |  |
|  | Mask |  |  | |  |
| Uncorrected | 4.77 | 0.17, 0.41 | <.001* | | 0.6 |
| Corrected | 4.88 | 0.17, 0.42 | <.001* | | 0.61 |
|  | No mask |  |  | |  |
| Uncorrected | 2.99 | 0.06, 0.28 | .004* | | 0.37 |
| Corrected | 3.17 | 0.06, 0.29 | .004* | | 0.4 |

*Note.* * marks statistically significant results. Results are shown separately for uncorrected and corrected data.

**Table S5.5**

*Memory for Face Identities (Question 1).* t *Tests Comparing the Sensitivity (d’) and Response Bias (c) of Familiar and Unfamiliar Faces*

|  | *T* | *95% CI* | *p_adj_* | *d* | |
| --- | --- | --- | --- | --- | --- |
| Sensitivity (d’) |  |  |  |  | |
| Uncorrected | 9.24 | 0.44, 0.68 | <.001* | | 1.16 |
| Corrected | 6.95 | 0.3, 0.53 | <.001* | | 0.87 |
|  |  |  |  | |  |
| Response Bias |  |  |  | |  |
| Uncorrected | -9.11 | -0.52, -0.33 | <.001* | | -1.14 |
| Corrected | -8.6 | -0.5, -0.31 | <.001* | | -1.08 |
|  |  |  |  | |  |

*Note.* * marks statistically significant results. Results are shown separately for uncorrected and corrected data.

**Table S5.6**

*Memory for Masked and Unmasked Faces (Question 1).* t *Tests Comparing the Sensitivity (d’) and Response Bias (c)*

|  | *t* | *95% CI* | *p_adj_* |  | | *d* | |
| --- | --- | --- | --- | --- | --- | --- | --- |
| Sensitivity (d’) |  |  |  |  | |  | |
| Uncorrected | -6.26 | -0.33, -0.17 |  | | <.001* | | -0.78 |
| Corrected | -6.32 | -0.32, -0.16 |  | | <.001* | | -0.79 |
|  |  |  |  | |  | |  |
| Response Bias |  |  |  | |  | |  |
| Uncorrected | 6.26 | 0.08, 0.16 |  | | <.001* | | 0.78 |
| Corrected | 6.32 | 0.08, 0.16 |  | | <.001* | | 0.79 |
|  |  |  |  | |  | |  |

*Note.* * marks statistically significant results. Results are shown separately for uncorrected and corrected data.

**Figure S5**

*Sensitivity (d’) and Response Bias (c) for Identity Recognition in Corrected Data (Question 1).*


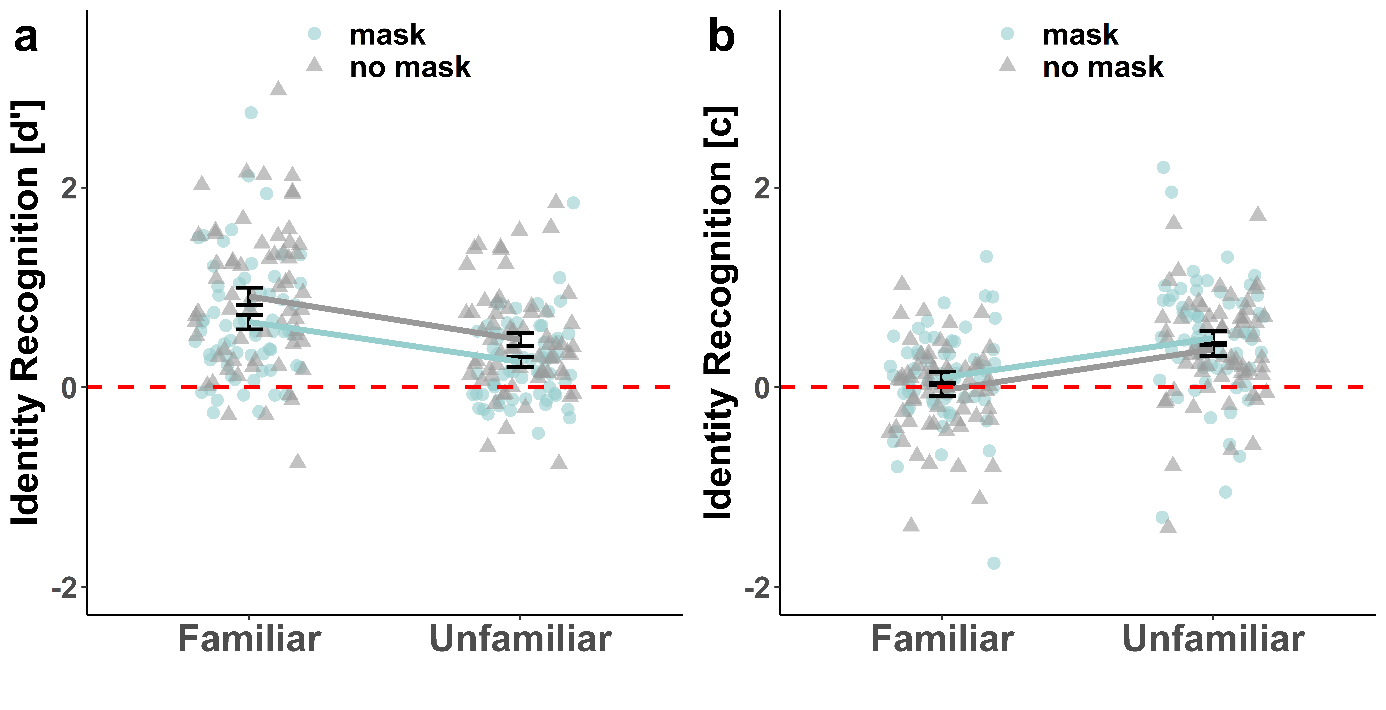


Note. Error bars indicate standard errors. Each datapoint shows the sensitivity (d’; panel a) or response bias (c; panel b) of a single participant indicating whether they saw a face in the study phase, separately for familiar and unfamiliar faces and faces that wore a mask (blue dots) or not (grey dots) in the study phase.

a) Main effects of familiarity and masks on recognition sensitivity for faces. Higher values of d’ indicate better performance. A value of zero indicates ‘guessing’.

b) Main effects of familiarity and masks on recognition bias for faces. Values greater than zero indicate a conservative bias (tendency to answer ‘no’) and values lower than zero indicate a liberal bias (tendency to answer ‘yes’).

Overall, these results are consistent with those reported in the section for the results of Question 1 (memory for identities) of Experiment 4. Participants showed a significant memory advantage (higher sensitivity) for familiar faces compared to unfamiliar faces (cf. Table S5.5; Figure S5a) and unmasked compared to masked faces (cf. Table S5.6). Participants also showed a significantly stronger conservative bias (tendency to indicate no memory) for unfamiliar than familiar faces (cf. Table S5.5; Figure S5b) and for masked compared to unmasked faces (cf. Table S5.6).

**Additional file 1: Data Figure S6**

**Figure S6**

*Frequency Plot for Trials, Separated by Conditions*
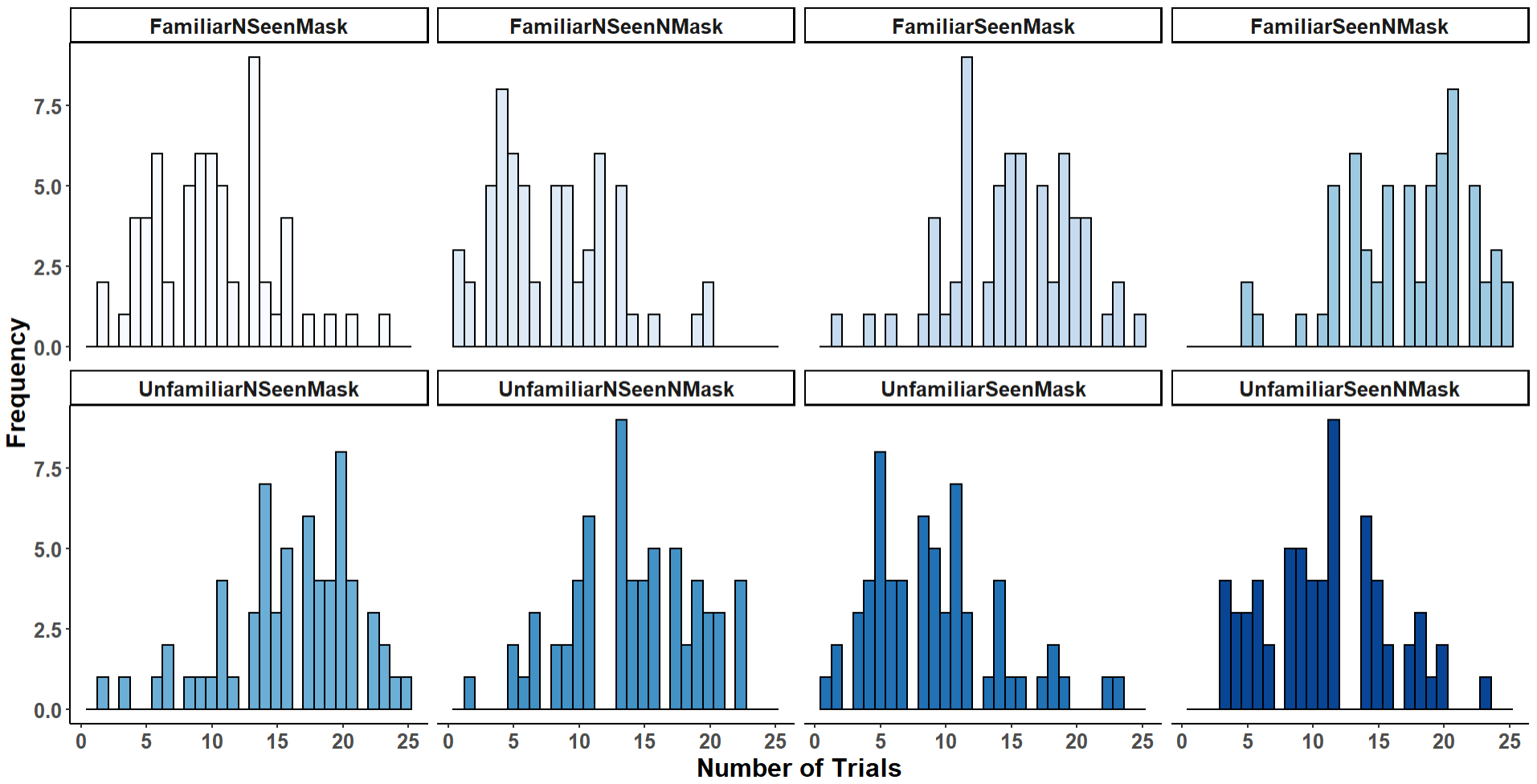


*Note*. For instance, ‘FamiliarNSeenNMask’ means that in this condition, participants indicated to not have seen the familiar face (Question 1) and it did not wear a mask in the study phase. For many participants the trial number was below a minimum of five trials for at least one of the conditions.

**Additional file 1: Analysis S7**

The analysis of Experiment 4 excluded nearly half of the participants (33 of 64) because they had fewer than 5 trials in at least one cell (cf. Additional file 1: Material S6). We set this criterion to ensure a minimum data basis for signal detection estimates for each of the eight resulting cells, crossing seen and unseen with familiar and unfamiliar as well as masked and unmasked faces. Including cases with very low trial numbers inevitably leads to extreme results for a given cell and participant (e.g. hit rates of 0 or 1), which have to be adjusted for signal detection analysis (cf. Methods). Moreover, extremely low numbers of trials will be more strongly biased by this kind of adjustment (cf. equations 4 and 5 in Methods). Nevertheless, here we investigated if our observed effects are robust when using a more liberal inclusion criterion of a minimum of 2 trials in each cell.

Descriptive statistics for d’ and c for answers to Question 2 (“Suppose this face was presented in the first part of the experiment. Was it shown with or without a mask?”) are reported in Table S7.1. We performed repeated measures 2x2 ANOVAs for the measures d’ and c respectively. The factors were familiarity (familiar vs. unfamiliar) and rating for faces (seen vs. not seen). For d’, the main effect of rating was significant as well as the interaction between both factors, familiarity and rating (see Table S7.2). For c, the main effect of rating was significant. Neither the main effect of familiarity nor the interaction between both factors were significant (see Table S7.2).

**Table S7.1**

*Memory for Masks (Question 2). Descriptive Statistics [mean(standard error of the mean)] for the Measures Sensitivity (d’) and Response Bias (c)*

|  | | Sensitivity (d‘) | | Response Bias (c) | |  |  |
| --- | --- | --- | --- | --- | --- | --- | --- |
|  | Familiar | | Unfamiliar | Familiar | Unfamiliar | | |
| Min. 5 Trials: |  | |  |  |  | | |
| Rated as seen | 0.65(0.14) | | -0.03(0.12) | 0.16(0.09) | 0.09(0.12) | | |
| Rated as not seen | -0.18(0.14) | | 0.17(0.07) | -0.08(0.20) | -0.02(0.22) | | |
|  |  | |  |  |  | | |
| Min. 2 Trials: |  | |  |  |  | | |
| Rated as seen | 0.74(0.09) | | 0.18(0.09) | 0.11(0.07) | -0.42(0.16) | | |
| Rated as not seen | -0.12(0.08) | | 0.12(0.06) | -0.36(0.14) | -0.02(0.08) | | |

*Note.* Results are shown separately for familiarity and cases in which participants rated the faces as seen or not seen in the study phase and (Question 1). Additionally, the results are listed separately for two differently selected criteria. Either the criterion was a minimum of 5 trials in each cell or a minimum of 2 trials.

**Table S7.2**

*Memory for Masks (Question 2). Repeated Measures ANOVA for Sensitivity (d’) and Response Bias (c)*

|  | Sensitivity (d‘) | Response Bias (c) |  |  |  |
| --- | --- | --- | --- | --- | --- |
| Min. 5 Trials |  |  |  |  |  |
| Familiarity | *F*(1, 30) = 2.67, *p* = .112, η_p_^2^ = .02 | *F*(1, 30) = 0.0, *p* = .953, η_p_^2^ = .00 | |  |  |
| Rating | *F*(1, 30) = 7.87, *p* = .009*, η_p_^2^ = .07 | *F*(1, 30) = 0.57, *p* = .455, η_p_^2^ = .01 | |  |  |
| Interaction | *F*(1, 30) = 21.91, *p* < .001*, η_p_^2^ = .16 | *F*(1, 30) = 1.48, *p* = .233, η_p_^2^= .00 | |  |  |
|  |  |  | |  |  |
| Min. 2 Trials: |  |  | |  |  |
| Familiarity | *F*(1, 55) = 3.58, *p* = .064, η_p_^2^ = .02 | *F*(1, 55) = 3.54, *p* = .065, η_p_^2^ = .00 | |  |  |
| Rating | *F*(1, 55) = 30.32, *p* < .001*, η_p_^2^ = .12 | *F*(1, 55) = 7.71, *p* = .007*, η_p_^2^ = .06 | |  |  |
| Interaction | *F*(1, 55) = 25.25, *p* < .001*, η_p_^2^ = .10 | *F*(1, 55) = 0.52, *p* = .475, η_p_^2^= .00 | |  |  |

*Note.* * marks statistically significant results. The results are listed separately for two differently selected criteria. Either the criterion was a minimum of 5 trials in each cell or a minimum of 2 trials.

Participants were significantly better in distinguishing whether a familiar face was wearing a mask (answer to Question 2) compared to unfamiliar ones, when they indicated they saw the face in the study phase (Question 1), *t*(55) = 4.46, *p_adj_* < .001, *d* = 0.6. There was a significant effect of better mask memory for unfamiliar faces compared to familiar ones, when rated as not seen, *t*(55) = -2.36, *p_adj_* = .044, *d* = -0.32. In addition, participants were better in distinguishing whether a familiar face wore a mask, when they rated the face as seen in the study phase compared to when they rated it as not seen, *t*(55) = -6.74, *p_adj_* < .001, *d* = -0.9. There was no difference between ratings for unfamiliar faces, *t*(55) = -0.59, *p_adj_* = .558, *d* = -0.08 (Figure S7a). Additionally, Table S7.3 summarizes the comparisons of the measure d’ with zero, depending on the different factor levels. Participants’ mask memory was significantly better than chance only for faces that were *both*, familiar and rated as seen. Overall, the effects for sensitivity are consistent with those reported for Experiment 4 and a set criterion of at least 5 trials. A difference was observed for the response bias. We observed no significant bias when we set the criterion to a minimum of five trials. However, when we set the criterion to a minimum of 2 trials, there was a significant liberal response bias to indicate that familiar and unfamiliar faces wore a mask when the faces were rated as not seen, *t*(55) = -2.78, *p* = .007, *d* = -0.37. In general, these results need to be treated with caution because some cells contain data from only a few trials (cf. Additional file 1: Material S6; cell: ‘FamiliarNSeenMask’) and this risks biases in signal detection analyses.

**Table S7.3**

*Memory for Masks (Question 2). One Sample* t *Tests Comparing the Sensitivity (d’) with Zero*

|  | Familiar | |  | |  |  |
| --- | --- | --- | --- | --- | --- | --- |
|  | *t* | *95% CI* | *p_adj_* | *d* | |  |
| Rated as seen | 7.83 | 0.55, 0.92 | <.001* | | 1.05 |  |
| Rated as not seen | -1.5 | -0.28, 0.04 | .138 | | -0.2 |  |
|  | Unfamiliar |  |  | |  |  |
| Rated as seen | 2.0 | 0.0, 0.36 | .136 | | 0.27 |  |
| Rated as not seen | 2.05 | 0.0, 0.24 | .136 | | 0.27 |  |

*Note.* * marks statistically significant results. Results are shown separately for cases in which participants rated the faces as seen or not seen in the study phase (Question 1).

**Figure S7**

*Sensitivity (d’) and Response Bias (c) for Mask Memory (Question 2)*


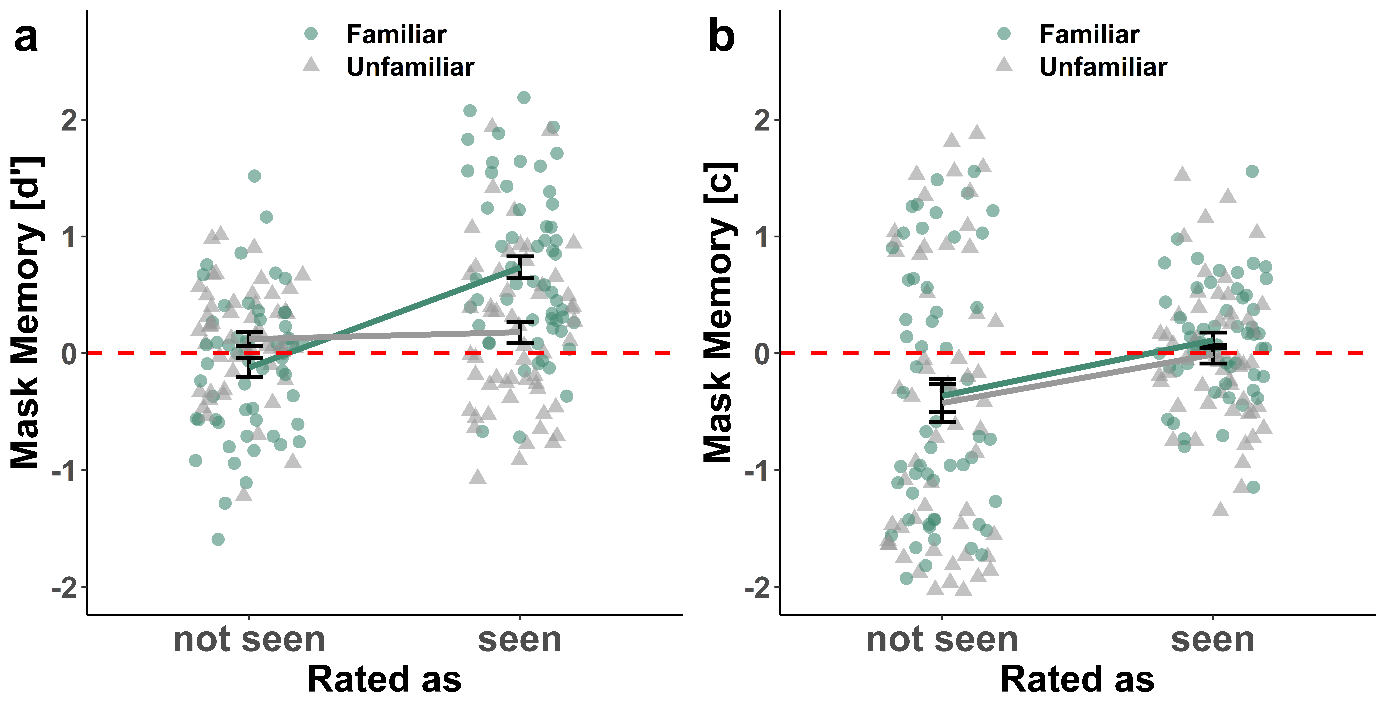


Note. Error bars indicate standard errors. Each datapoint shows the sensitivity (d’; panel a) or response bias (c; panel b) of a participant indicating whether they saw a mask in the study phase, separately for cases in which they rated the faces as seen or not seen in the study phase and familiar (blue dots) or unfamiliar faces (grey dots).

a) Main effects of familiarity and rating on memory sensitivity for masks. Higher values of d’ indicate better performance. A value of zero indicates ‘guessing’.

b) Main effects of familiarity and rating on memory bias for masks. Values greater than zero indicate a conservative bias (tendency to answer ‘no’) and values lower than zero indicate a liberal bias (tendency to answer ‘yes’).
